# Dynamic laminar rerouting of inter-areal mnemonic signal by cognitive operations in primate temporal cortex

**DOI:** 10.1038/s41467-018-07007-1

**Published:** 2018-11-06

**Authors:** Masaki Takeda, Toshiyuki Hirabayashi, Yusuke Adachi, Yasushi Miyashita

**Affiliations:** 1 Department of Physiology, The University of Tokyo School of Medicine, 7-3-1 Hongo, Bunkyo-ku, Tokyo, 113-0033 Japan; 2 Juntendo University Graduate School of Medicine, 2-1-1 Hongo, Bunkyo-ku, Tokyo, 113-8421 Japan; 3 Research Center for Brain Communication, Kochi University of Technology, Kami-city, Kochi 782-8502 Japan

## Abstract

Execution of cognitive functions is orchestrated by a brain-wide network comprising multiple regions. However, it remains elusive whether the cortical laminar pattern of inter-areal interactions exhibits dynamic routings, depending on cognitive operations. We address this issue by simultaneously recording neuronal activities from area 36 and area TE of the temporal cortex while monkeys performed a visual cued-recall task. We identify dynamic laminar routing of the inter-areal interaction: during visual processing of a presented cue, spiking activities of area 36 neurons are preferentially coherent with local field potentials at the supragranular layer of area TE, while the signal from the same neurons switches to target the infragranular layer of area TE during memory retrieval. This layer-dependent signal represents the to-be-recalled object, and has an impact on the local processing at the supragranular layer in both cognitive operations. Thus, cortical layers form a key structural basis for dynamic switching of cognitive operations.

## Abstract (web summary)

Inter-areal interaction has been shown to support various cognitive functions. Here, the authors report that neurons in area 36 flexibly synchronize their activity with different layers of area TE within different epochs of a visually cued recall task suggesting dynamic rerouting of information.

## Introduction

**Fig. 1:**
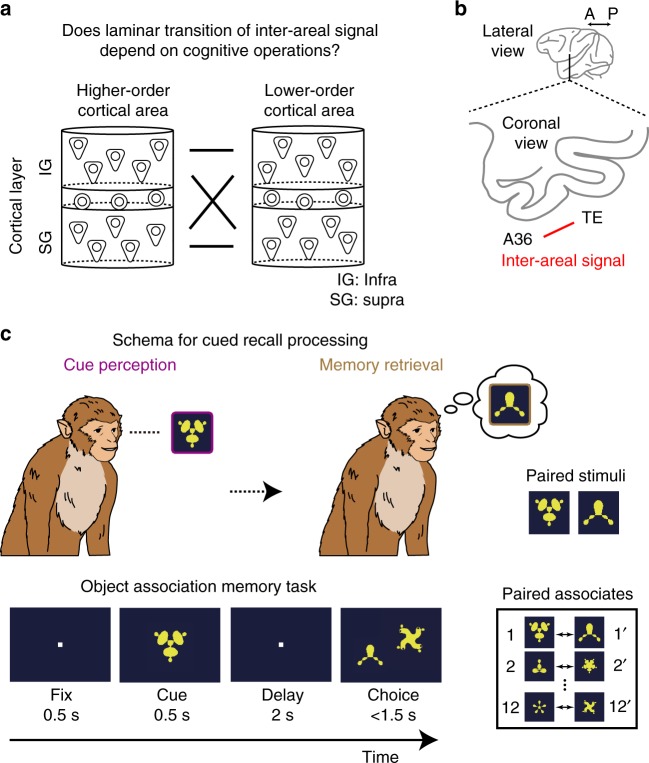
Schematic models of cortical laminar transition of the inter-areal signal. **a** It remains elusive whether the laminar transition of inter-areal signal between higher-order and lower-order cortical areas depends on cognitive operations. IG infragranular layer, SG supragranular layer. **b** Lateral and coronal views of a monkey brain. A36, area 36. TE, area TE. A, anterior. P, posterior. **c** Behavioral task. To characterize laminar specificity of inter-areal interaction during cue perception and memory retrieval in cued-recall, an object association memory task was used. In the task, monkeys were required to retrieve the learned paired associate of the presented cue stimulus. See Methods for details

Execution of various cognitive functions is orchestrated by a brain-wide neuronal network^1–4^. Several lines of evidence indicate that distinct brain regions cooperate via coherent activities^5,6^ in various frequency ranges for object perception^7^, associative learning^8^, short-term memory^9,10^, working memory^11,12^, attention^6,13–16^, and decision making^17^. Recently, accumulating electrophysiological evidence has suggested that the coordination between distinct brain regions, such as cortico-cortical^18^ and cortico-limbic^19–21^, varied depending on the cognitive operations in which subjects were engaged. For example, phase-locking of spiking activity in the rat medial prefrontal cortex to the gamma activity of the local field potential (LFP) in the hippocampus has been shown to be stronger during the encoding phase than during the retrieval phase of spatial working memory^19^. Such cognitive-state-dependent changes in functional connectivity between brain regions have also been demonstrated by imaging studies in humans^22–24^ and non-human primates^25–27^. However, these previous studies did not distinguish neuronal activities in different cortical layers, even though it is well known that functional and anatomical segregation exists across the layers^28–30^. Thus, the underlying laminar basis for the dynamic flexibility of inter-areal signals remains unknown (Fig. 1a).

Neuronal coordination between subregions of the inferior temporal (IT) cortex, i.e., area 36 (A36) and area TE (TE) (Fig. 1b), is a candidate for the cognitive operation-dependent laminar routing of inter-areal signals, for the following reasons. First, A36 and TE are known as the pivotal point where the ventral visual pathway and the medial temporal lobe memory system intersects^1,3,4,31,32^ and these areas are engaged in cued-recall involving multiple cognitive operations, i.e., perception of the presented cue and retrieval of memory with the help of the cue. Converging evidence, particularly electrophysiological evidence, has suggested that A36 and TE engage in the associative memory of visual objects^33–41^; memory neurons have been identified whose activities encode the presented objects (cue-holding neuron) and the to-be-recalled objects (pair-recall neurons), as well as association of the presented objects and to-be-recalled objects (pair-coding neurons). Second, it has recently been demonstrated that the backward signal from A36 to TE has an impact on inter-laminar processing in TE during successful retrieval of object association memory^42^. Thus, the inter-areal neuronal circuit between A36 and TE is of interest for gaining insight into the dynamic modulation of laminar routing between brain regions according to cognitive operations.

We hypothesized two models for the dynamics of inter-areal laminar flow between A36 and TE in the cued-recall of an object association memory. In the static flow model, the activity of an A36 neuron is always coordinated with the activity in a specific layer of TE (e.g., infragranular or supragranular layer) during both cue perception and memory retrieval, and different populations of A36 neurons whose activities are coordinated with the activities in different layers of TE are separately engaged in the cue perception and the memory retrieval. Alternatively, in the dynamic flow model, a given A36 neuron is engaged in both cue perception and memory retrieval, and the layer of TE showing coordinated activity with the A36 neuron is dynamically switched, depending on the stages of cue perception and memory retrieval. In the present study, we tested these hypotheses by simultaneously recording activities of single neurons in A36 and LFPs in each layer of TE of the temporal cortex while monkeys were performing a visual cued-recall task (Fig. 1c), and found that the cortical laminar pattern of inter-areal interactions between A36 and TE exhibits dynamic routings, depending on cognitive operations.

## Results

### Inter-areal spike-LFP coherence during cue period

**Fig. 2:**
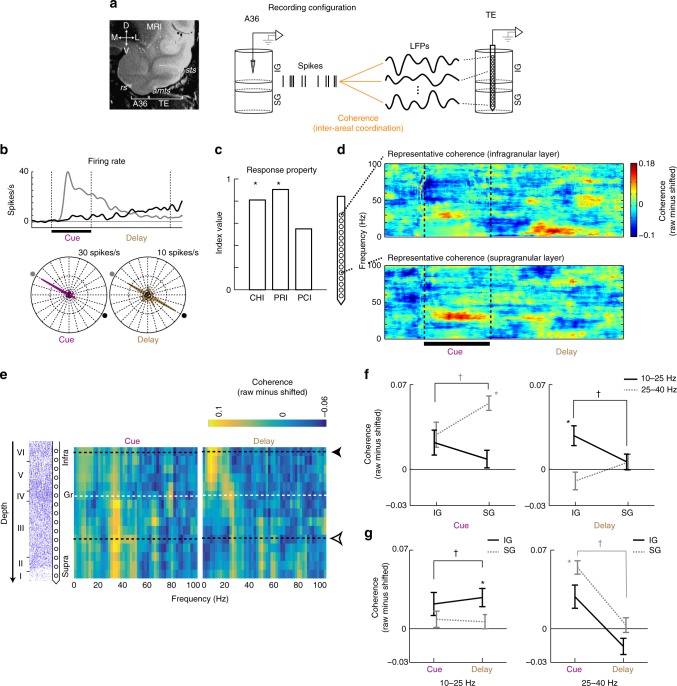
Recording configuration and representative data showing dynamic transition in layer specificity of the inter-areal signal. **a** Spiking activities were recorded from A36, while local field potentials (LFPs) were recorded from TE by using a multi-contact linear array electrode. Inter-areal interaction was examined by coherence between these two signals. Cortical depths of the recording sites in A36 and TE were determined by histology and current source density of the LFP (see also Supplementary Fig. 13). IG infragranular layer, SG supragranular layer. **b**–**f** Representative data. **b** Top, spike density functions for the trials in which the presented cue stimulus exerted the largest response during the cue period (gray) and its paired associate was presented as a cue stimulus (black). We analyzed the latter trial (shown in black) for inter-areal coherence. Bottom, polar plots of mean firing rates during the cue and delay periods, shown for each cue stimulus. The responses to a stimulus and its paired-associate are indicated by radial lines. **c** Stimulus coding indices. CHI cue-holding index, PRI pair-recall index, PCI pair-coding index. *, *P* < 0.05. **d** Dynamics of coherence (coherogram) between A36 spikes and TE LFPs at the infragranular layer (top) and at the supragranular layer (bottom). The subtraction of trial-shifted control from raw coherence is shown. **e** Layer specificity of coherence as a function of frequency. The amplitude of coherence is color-coded. Black and white arrowheads depict coherograms shown in **d**. Left, corresponding histological section. **f** Laminar dynamics of coherence at 10−25 (black solid line) and 25−40 (gray dotted line) Hz during the cue (left) and delay (right) periods. **g** Temporal dynamics of coherence in IG and SG Hz at 10−25 to 25−40 Hz. *P* < 0.0001 for interaction between layer and frequency, between period and layer, and between period and frequency; *F* = 0.15, *P* = 0.69 for interaction among three effects, by three-way ANOVA for the effects of period, layer and frequency. *, *P* < 0.05; *t*-test against zero, corrected for multiple comparisons. ^†^, *P* < 0.05; paired *t*-test, corrected for multiple comparisons. Error bars, mean ± s.e.m.

Two macaque monkeys were trained to perform the visual cued-recall task^33–40,42,43^. Neuronal signals from A36 (spiking activity) and TE (LFP) were recorded using tungsten electrodes and multi-contact linear electrodes (16 channels with 150 μm spacing), respectively (Fig. 2a). For each A36 neuron, we first determined the optimal cue stimulus that evoked (1) the strongest delay activity and (2) the top-four cue activity among the 24 stimuli. We then defined the optimal trial as all trials presenting the above determined optimal cue stimulus. We analyzed the inter-areal coherence between A36 spikes and TE LFPs in the optimal trials (see Methods for details).

First, we examined the inter-areal signal between A36 and TE by using spike-field coherence, which involves directed synaptic influence^11,17,42^ from spiking activities to LFPs, as well as the confluence of signals from local and distal regions. During the cue period, spiking activities in A36 were coherent with LFPs recorded by at least one channel of the electrode in TE (Supplementary Fig. 1 for representative data; Supplementary Fig. 2 for population data). Coherence during the cue period was significantly higher than that of the trial-shifted control in the frequency range lower than 40 Hz, with the spectral peak at around 10−20 Hz (Supplementary Fig. 2b; paired *t*-test, *P* < 0.01, corrected for multiple comparisons across frequencies by Bonferroni’s method; *n* = 56 datasets). The amplitude of coherence in this frequency range was dependent on the presented cue stimulus (Supplementary Fig. 2c; one-way ANOVA: *F* = 18.7, *P* = 8.21 × 10^−8^, followed by the post-hoc Tukey−Kramer test, *P* < 0.001). The distribution of the mean angular phase at 10‒25 Hz was significantly concentrated (Supplementary Fig. 2d; Rayleigh test: *z* = 3.69, *P* < 0.0244), suggesting consistent phase-locking of spikes on LFPs.

### Dynamic laminar routing of inter-areal coherence

A previous study demonstrated that, during the delay period, the inter-areal coherence between A36 spikes and TE LFPs has a layer specificity in TE^42^. Is the inter-areal coherence during the cue period also layer specific, and if so, is the layer specificity of the coherence consistent between the cue and the delay periods? Figure 2b−g shows representative data depicting the dynamic layer transition of inter-areal coherence between the cue and the delay periods. The channel at the granular layer (Gr) in TE was estimated based on the current source density (CSD) calculated from the depth profile of visually evoked LFPs^42–44^. The A36 neuron preferentially responded to the optimal stimulus during the cue period, and, the activity was retained during the subsequent delay period. When the paired associate of the optimal stimulus was presented, the firing rate gradually increased during the delay period, representing the retrieval signal of the optimal stimulus (Fig. 2b, c). The spiking activity of this A36 neuron depicted temporally dynamic coherence with the LFP in TE (Fig. 2e–g; three-way ANOVA for the effects of period, layer and frequency: *P* < 0.0001 for interaction between layer and frequency, between period and layer, and between period and frequency; *F* = 0.15, *P* = 0.69 for interaction among three effects). During the cue period, the spiking activity in A36 was coherent with the LFP at the supragranular layer (SG) of TE (Fig. 2d, e). Notably, during the delay period, the inter-areal coherence disappeared in SG, but emerged in the infragranular layer (IG) of TE. The frequency of the coherence also changed from 25 to 40 Hz (*γ* frequency) during the cue period to a lower frequency range (10−25 Hz; *β* frequency) during the delay period. As a result, significant coherence was observed in SG during the cue period at 25‒40 Hz (Fig. 2f, g; *t* = 9.10, *P* = 5.08 × 10^−11^, paired *t*-test against zero corrected for multiple comparisons by Bonferroni’s method; difference in coherence between cue and delay in SG at 25‒40 Hz: *F* = 30.92, *P* < 0.001) and in IG during the delay period at 10‒25 Hz (*t* = 3.38, *P* = 0.0024, paired *t*-test against zero corrected for multiple comparisons by Bonferroni’s method; difference in coherence between cue and delay in IG at 10‒25 Hz: *F* = 5.30, *P* = 0.023). Another representative data in which the A36 neuron coded both the optimal cue and its paired associate during the cue and delay periods is shown in Supplementary Figure 3.

**Fig. 3:**
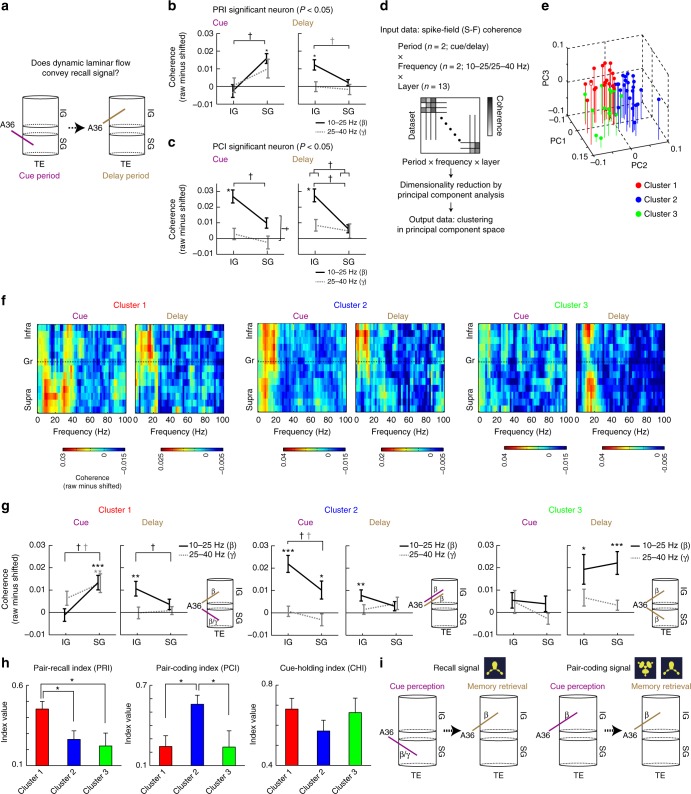
Population layer specificity of coherence during cue and delay periods. **a** Schematic drawing of dynamic laminar flow conveying target recall signal. **b**, **c** Laminar pattern of coherence (mean ± s.e.m.) with neurons showing significant signal contents [pair-recall index (PRI)/pair-coding index (PCI)]. *, *P* < 0.05; comparison with zero by paired *t*-test corrected for multiple comparisons. ^†^, *P* < 0.05; main effect or interaction in ANOVA. **d** Diagram for estimation of laminar transition of inter-areal coherence. Coherence profiles (two periods × two frequency ranges × 13 layers for each dataset) were classified into clusters according to k-means clustering in the principal component (PC) space. See Methods and Supplementary Figure 5a for details. **e** Scatter plot for first three PC values of individual datasets. *n* = 56. Each dataset is color-coded for the clusters determined by k-means clustering (red = cluster 1, blue = cluster 2, green = cluster 3; note that red open circle depicts data in Fig. 2). **f** Population dynamics of layer specificity in coherence (ordinate) from the cue (left) to the delay (right) periods, as a function of frequency (abscissa). *n* = 19, 24, and 13 for clusters 1, 2, and 3, respectively. **g** Laminar dynamics of coherence (mean ± s.e.m.) at 10−25 (black solid line) and 25−40 (gray dotted line) Hz from the cue (left) to the delay (right) periods. *, *P* < 0.05; **, *P* < 0.01; and ***, *P* < 0.001; comparison with zero by paired *t*-test corrected for multiple comparisons. ^†^, *P* < 0.05; paired *t*-test corrected for multiple comparisons. Right inset for each cluster summarizes the layer specificity of inter-areal coherence during the cue (brown) and the delay (purple) periods. **h** Signal content of A36 neurons in each cluster (mean ± s.e.m.). *, *P* < 0.05; Tukey−Kramer test after one-way ANOVA. **i** Summary schema for laminar transition of inter-areal coherence for target recall (cluster 1) and pair-coding (cluster 2) signals. IG infragranular layer, SG supragranular layer

We then examined the relationship between signal contents of A36 neurons and the layer specificity of coherence between A36 and TE (Fig. 3a). For each neuron, neuronal coding of memory representation was quantified by the indices that extracted the response components coding for the to-be-recalled object (pair-recall index, PRI), the pairing between the presented cue and the to-be-recalled object (pair-coding index, PCI), as well as the presented cue (cue-holding index, CHI) (see Methods for details). When selecting datasets showing a significant PRI value at a threshold of *P* < 0.05 (Fig. 3b, Supplementary Fig. 4a), we found a significant interaction between time-period and layer effects (*F* = 30.34, *P* = 0.0004) at 10–25 Hz and a marginally significant interaction (*F* = 3.90, *P* = 0.080) and period effect (*F* = 3.75, *P* = 0.085) at 25–40 Hz (Supplementary Fig. 4a), as well as a significant layer effect during the cue and delay periods (Fig. 3b; *F* = 11.58, *P* = 0.0078 for cue; *F* = 5.54, *P* = 0.043 for delay). We also selected datasets showing a significant PCI value at *P* < 0.05, and then tested the effects as we did for those with a significant PRI value (Fig. 3c, Supplementary Fig. 4b). The datasets with significant PCI values showed a significant layer effect (*F* = 49.35, *P* = 0.0002) and marginally significant interaction (*F* = 3.86, *P* = 0.090) at 10−25 Hz (Supplementary Fig. 4b). A two-way ANOVA for layer and frequency revealed a significant layer effect (*F* = 8.85, *P* = 0.021) and frequency effect (*F* = 13.86, *P* = 0.0074) during the cue period, as well as a significant layer effect (*F* = 34.11, *P* = 0.0006) and interaction (*F* = 26.20, *P* = 0.0014) during the delay period (Fig. 3c).

To examine the dynamics of the layer specificity of inter-areal coherence at the all population level (*n* = 56), we employed the data-driven clustering of all datasets using an unsupervised approach as follows. We first extracted principal components (PCs) of coherence during both the cue and delay periods at the frequency ranges of 10−25 Hz (*β*) and 25−40 Hz (*γ*) (coherence profiles) (Fig. 3d, Supplementary Fig. 5a, b; see Methods for detailed procedures). In the PC space, we next classified all datasets into clusters. By using several statistical procedures for determining the optimal number of clusters, we found three clusters showing distinct transition of the layer specificity of coherence from the cue to the delay periods (Fig. 3e−g; Supplementary Fig. 5c−d, 6, 7 and 8; see Supplementary Fig. 9 for individual coherence profiles; see Methods for details). The three clusters depicted significantly different patterns of coherence profiles [four-way ANOVA for clusters (1−3), layers (IG/SG), periods (cue/delay), and frequencies (10−25/25−40): *F* = 3.48, *P* = 0.0381 for interaction between clusters and layers; *F* = 12.4, *P* < 0.0001 for interaction between clusters and periods; and *F* = 5.54, *P* = 0.0065 for interaction between clusters and frequencies; see also Supplementary Fig. 10a−d for coherence profiles in each cluster of each monkey]. In cluster 1 (*n* = 19), significant coherence was predominantly observed in SG during the cue period at both *β* and *γ* frequency ranges [Fig. 3g; paired *t*-test against trial-shifted control: *t* *=* 5.65, *P* = 4.64 × 10^−5^ (*β*) and *t* = 3.29, *P* = 0.00800 (*γ*), corrected for multiple comparisons by Bonferroni’s method]. In contrast, during the delay period, significant coherence was observed in IG only at the *β* frequency (*t* = 3.23, *P* = 0.00940). We found a significant interaction between task time-period (cue/delay) and layer (IG/SG) in cluster 1 (*F* = 25.22, *P* < 0.0001 at 10−25 Hz; Supplementary Fig. 8). In cluster 2 (*n* = 24), coherence in IG at the *β* frequency remained significant throughout the cue and delay periods (Fig. 3g; *t* = 5.76, *P* = 1.46 × 10^−5^ and *t* *=* 3.10, *P* = 0.0108 for the cue and delay periods, respectively). In cluster 3 (*n* = 13), significant coherence was observed only during the delay period in both IG and SG at the *β* frequency (Fig. 3g; *t* = 2.91, *P* = 0.0262 and *t* = 4.34, *P* = 1.92 × 10^−3^ for IG and SG, respectively). These results suggest that, in cluster 1, the directed layer of the inter-areal signal dynamically changes between the stages of cue perception and memory retrieval. The overall coherence in TE did not show significant differences between layers or periods (Supplementary Fig. 11; two-way ANOVA for period and layer effects), suggesting that such laminar rerouting of coherence is not a general occurrence. Note that, as theoretically expected, it is unlikely that either LFP power or spike power influenced the spike-field measurements, which differed between clusters (Supplementary Fig. 12).

We next examined the relationship between the signal contents conveyed by the activity of A36 neurons and the laminar dynamics of inter-areal coherence in each cluster (Fig. 3h). A36 neurons in cluster 1 more predominantly encoded the to-be-recalled object than neurons in clusters 2 or 3 [*P* < 0.05, Tukey–Kramer test after one-way ANOVA (*F* = 4.21, *P* = 0.0200)]. In contrast, neurons in cluster 2 more predominantly encoded both the presented cue and the to-be-recalled object than neurons in clusters 1 and 3 [*P* < 0.05, Tukey–Kramer test after one-way ANOVA (*F* = 5.26, *P* = 0.0083)]. Note that these results were consistent across monkeys [Supplementary Fig. 10e, f; three-way ANOVA (monkey effect × cluster effect × index effect): *F* = 0.0451, *P* = 0.833 for monkey effect; *F* = 1.13, *P* = 0.332 for monkey effect × cluster effect; *F* = 2.50, *P* = 0.0870 for monkey effect × index effect; and *F* = 1.55, *P* = 0.193 for monkey effect × cluster effect × index effect].

Taken together, these results suggest that the inter-areal mnemonic signal for the to-be-recalled object dynamically reroutes the directed layer in TE, depending on the processing stage (cluster 1), while the inter-areal signal for the pairing of objects is consistently directed to the infragranular layer of TE (cluster 2) (Fig. 3i).

In cluster 1, A36 neurons were located in a deeper part of the cortex than the granular layer (Supplementary Fig. 13; *z* = 2.37, *P* = 0.0352, Wilcoxon’s signed-rank test corrected for multiple comparisons with Bonferroni’s method; see Methods for detailed procedures). A36 neurons in cluster 2 were also located in a deeper part of the cortex than the granular layer, but this finding did not reach statistical significance (*P* = 0.0616). A similar tendency in the relative cortical depth of memory neurons in A36 has been reported previously: neurons coding the to-be-recalled object were preferentially located in layer 6, while neurons coding pairing of objects were preferentially located in layer 5^40^.

### Coupling between the inter-areal and inter-laminar signal

**Fig. 4:**
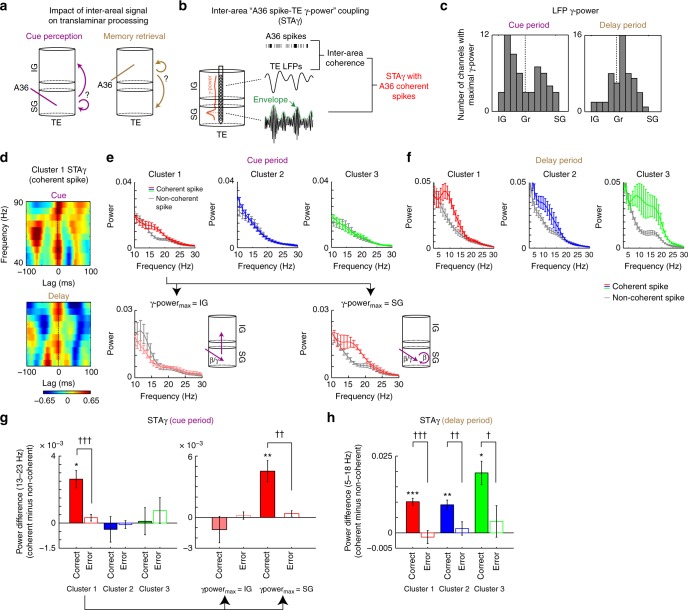
Impact of inter-areal coherence on inter-laminar signal processing in TE. **a** Schematics for cascades of inter-areal and inter-laminar signal processing. **b** Diagram of spike-triggered average (STA) of the γ-power (STAγ). A36 spikes that were coherent with TE local field potentials (LFPs) at the channel showing maximum coherence were first extracted (shown in black ticks) from all spike trains. Then, a STA of γ activity envelope (green) was calculated. The γ activity envelope was selected at the TE channel showing maximum γ-power (in this example, in SG). See Methods for details. **c** Histograms of TE channels showing maximum γ-power. Gr granular layer. *n* = 56. See also Supplementary Figure 14. **d** Population STAγ with coherent spikes in cluster 1. **e** Power spectrum of STAγ (STAγ power) for each cluster (mean ± s.e.m.) during the cue period. STAγ power with coherent spikes and non-coherent spikes are shown separately. *n* = 19, 24, and 13 for clusters 1, 2, and 3, respectively. Bottom panels depict the STAγ power for datasets with maximum γ-power at infragranular layer (IG, left) and at the supragranular layer (SG, right). **f** The STAγ power for each cluster during the delay period. See also Supplementary Figure 15. **g**, **h** Error analysis of STAγ for each cluster during the cue period (**g**) and the delay period (**h**). Shown are the mean (±s.e.m.) values of difference in STAγ power between coherent and non-coherent spikes at the frequency range of 13−23 Hz (cue) and 5−18 Hz (delay). *, comparison with zero (*, *P* < 0.05; **, *P* < 0.01; ***, *P* < 0.001; paired *t*-test corrected for multiple comparisons). ^†^, *t*-test corrected for multiple comparisons (^†^, *P* < 0.05, ^††^, *P* < 0.01, ^†††^, *P* < 0.001). Note that the number of correct trials was reduced to be matched with that of error trials (see Methods)

We hypothesized that, for both cue perception and memory retrieval, the translaminar signal processing in TE would be recruited by way of the inter-areal coherent network (Fig. 4a). To determine whether inter-areal coherence between the A36 spikes and the TE LFPs has an impact on local neuronal processing in TE during cue perception and memory retrieval (Fig. 4a), we examined the coupling between the inter-areal neuronal signal and the time-varying *γ* activity in TE. To this end, we employed A36 spikes that were involved in coherence with TE LFP (Fig. 4b). A36 spikes coherent with TE LFP at the channel showing maximum coherence (coherent spike; spikes firing at φ_max_ ± 1/4 *π*) were extracted to calculate the spike-triggered average (STA) of the TE γ-power (STAγ). In a previous study, STAγ successfully captured the impact of the inter-areal signal on inter-laminar signal processing^42^ [see Methods for detailed procedures; see also Discussion for functional difference between STAγ and other neuronal measurements, i.e., phase-amplitude coupling (PAC) and coherence]. The STAγ was calculated for the TE channel showing the maximum γ-power.

We first examined the layer specificity of the LFP power in the higher gamma frequency range (40−90 Hz, γ-power) as an index of local neuronal processing^5,42,45^ in TE. Compared with the fixation period, the γ-power during the cue period was significantly elevated in SG as well as IG (Supplementary Fig. 14a, b; paired *t-*test: *t* = 7.68, *P* < 0.0001 for SG; *t* = 10.8, *P* < 0.0001 for IG), which was reflected in the comparable number of datasets showing the maximum γ-power in IG and SG [Fig. 4c; *P* = 0.181, *χ*^2^ = 1.79; χ^2^ test for difference in the number of datasets showing the maximum γ-power between IG (*n* = 30) and SG (*n* = 23)]. During the delay period, in contrast, the γ-power was predominantly elevated in SG, but not in IG (Supplementary Fig. 14c, d; paired *t-*test: *t* = 1.13, *P* = 0.264 for IG; *t* = 6.15, *P* < 0.0001 for SG), which was reflected in the significant difference in the number of datasets showing the maximum γ-power [Fig. 4c; *P* = 0.00186, *χ*^2^ = 9.68; IG (*n* = 14) and SG (*n* = 36)]. These results were consistent with previous studies in which gamma-band synchrony in superficial layers increased with attention in the early visual areas^46^. Note that, in both the cue and delay period, the layer specificity of γ-power did not significantly differ among clusters (Supplementary Fig. 14; two-way ANOVA for clusters and laminar position: *F* = 0.787, *P* = 0.465 for cluster effect in the cue period, *F* = 1.05, *P* = 0.365 for cluster effect in the delay period).

Figure 4d depicts a population STAγ with coherent spikes in cluster 1, which showed a low-frequency periodic increase in γ-power during both the cue and delay periods. We examined the spectral properties of the STAγ for datasets in each cluster separately (Fig. 4e, f). During the cue period, the population STAγ (STAγ power) with coherent spikes in cluster 1 was greater than that calculated with non-coherent spikes, specifically in the low-frequency range (13−23 Hz, *β*), whereas no difference in STAγ power between coherent and non-coherent spikes was observed in clusters 2 and 3 (Fig. 4e). Interestingly, in cluster 1, the amplitude of STAγ power depended on the cortical layer of the maximum γ-power: the STAγ power for coherent spikes was greater than that for non-coherent spikes only in datasets showing the maximum γ-power in SG, but not in IG. In contrast, during the delay period, all clusters showed increased STAγ power at the 5−18 Hz (*α*) frequency range (in cluster 3, the frequency of increase in STAγ power extended to about 25 Hz; Fig. 4f). The increase in STAγ power during the delay period predominantly emerged when the SG channel showed the maximum γ-power (Supplementary Fig. 15).

Finally, we examined whether the observed increase in STAγ power in cluster 1 during the cue period was related to the monkey’s behavioral performance by comparing the STAγ power in correct trials with that in error trials. The difference in the STAγ power between coherent and non-coherent spikes was significantly greater in correct trials than in error trials, and was statistically significant only in correct trials [Fig. 4g, left; interaction of two-way ANOVA for clusters and performance (correct/error): *F* = 12.5, *P* < 0.0001; *t*-test between correct and error trials in cluster 1: *t* = 4.13, *P* = 9.15 × 10^−4^, corrected for multiple comparisons by Bonferroni’s method; *t*-test against zero corrected for multiple comparisons: correct trials in cluster 1, *t* = 2.54, *P* = 0.0420; error trials in cluster 1, *t* = 1.59, *P* = 0.226]. Furthermore, this behavioral impact was observed in the datasets where the maximum γ-power was observed in SG (Fig. 4g, right; *t*-test between correct and error trials: *t* = 3.80, *P* = 0.00240; *t*-test against zero: correct trials, *t* = 4.33, *P* = 0.00220; error trials, *t* = 1.43, *P* = 0.282), but not in IG (*t*-test between correct and error trials: *t* = 0.995, *P* = 0.686; *t*-test against zero: correct trials, *t* = -0.927, *P* = 0.792; error trials, *t* = 0.527, *P* = 0.654). During the delay period, all clusters showed a significant difference in the STAγ power between coherent and non-coherent spikes only in correct trials [Fig. 4h; two-way ANOVA for clusters and performance (correct/error): *F* = 37.4, *P* < 0.0001 for main effect of performance; *t*-test between correct and error trials: *t* = 4.67, *P* = 6.78 × 10^−5^, *t* = 4.13, *P* = 0.007, and *t* = 2.37, *P* = 0.027 in clusters 1, 2, and 3, respectively]. Note that the spiking activity in A36 (Supplementary Fig. 16a), the γ-power in TE (Supplementary Fig. 16b−c), the coherence between A36 and TE (Supplementary Fig. 16d−e), and the PAC in TE (Supplementary Fig. 17) showed no differences between correct and error trials. Taken together, these results suggest that the coupling between inter-areal and translaminar signal processing in cluster 1 during both the cue and delay periods can predict successful retrieval of long-term object memory.

## Discussion

**Fig. 5:**
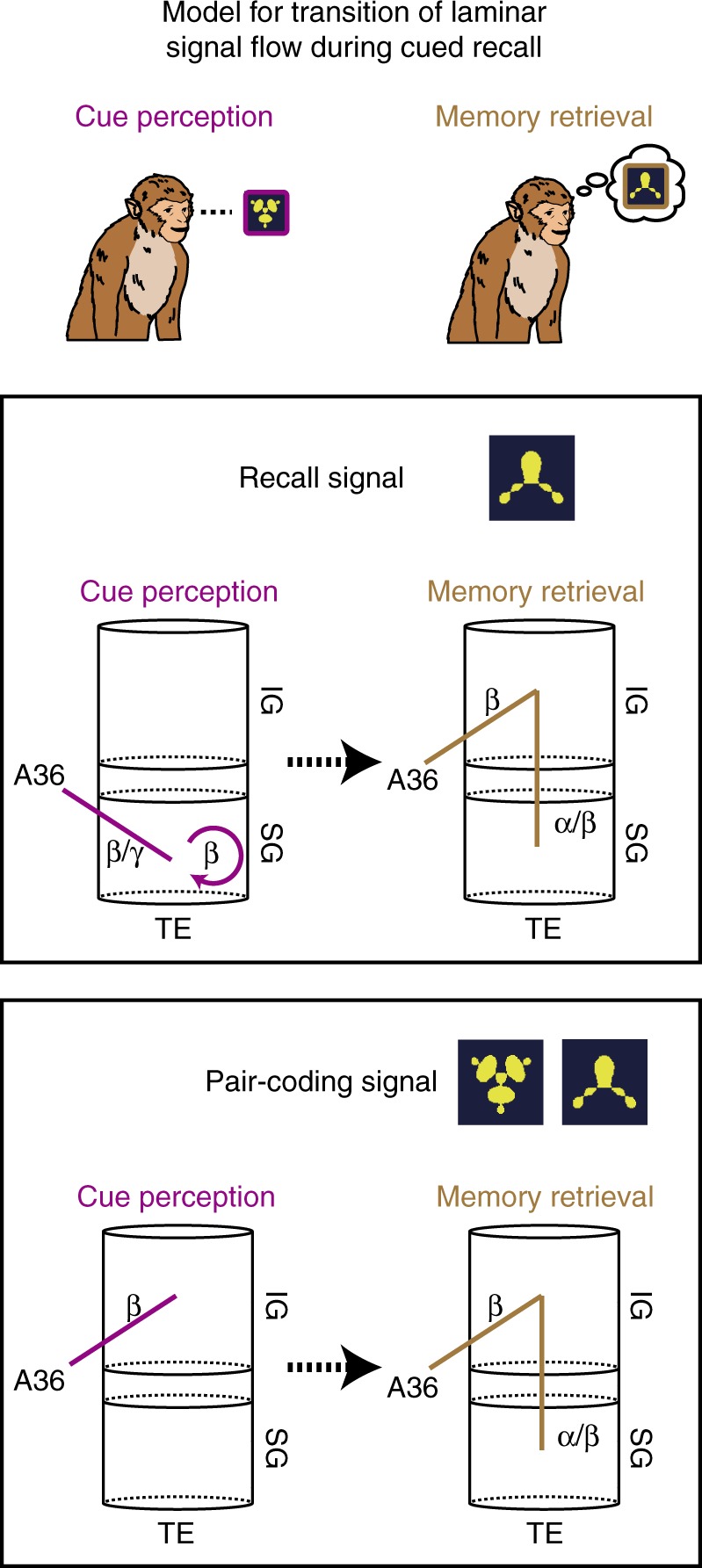
Summary model for laminar transition of signal flow for cued-recall. Schematic summary of signal flows in cluster 1 (top, recall signal) and 2 (bottom, pair-coding signal). Cluster 1: during the stage of cue perception, the inter-areal signal for to-be-recalled object emerges between A36 and the supragranular layer of TE in the higher frequency range (*β* and *γ*) and then has an impact on local signal processing at the supragranular layer (SG). During the memory retrieval stage, the inter-areal signal re-routes the directed layer to the infragranular layer (IG) of TE at *β* frequency, which then targets supragranular layer. Cluster 2: inter-areal signal conveying information of the pairing of stimuli targets the infragranular layer of TE throughout the stages of cue perception and memory retrieval

In this study, neuronal activities in A36 and TE were simultaneously recorded while monkeys performed a cued-recall task. We found that the inter-areal signal flow, which conveyed the information of the to-be-recalled object, dynamically switched the directed cortical layer in TE, in a manner dependent on the cognitive operations involved: supragranular and infragranular layers for cue perception and memory retrieval, respectively (Fig. 5). Moreover, this signal had an impact on the inter-laminar signal processing within TE only when monkeys successfully retrieved the sought object.

Using multi-contact linear-array electrodes, previous studies have demonstrated the layer specificity of feedforward signal flows between brain regions such as the flow from the lateral geniculate nucleus to the primary visual cortex^47^, from V1 to V2^48^, and from V1 to V4^49^. The laminar pattern of these feedforward signal flows was in line with the canonical anatomical projections that terminate at the granular layer of the target brain region^28–30,50^. In contrast, the laminar pattern of the backward signal directed to lower-order areas remains unclear. Anatomically, projection from a higher-order area shows a multi-laminar pattern that avoids the granular layer of the lower-order area^50^. Indeed, in the temporal cortex, four axonal arborization patterns of the backward projections have been morphologically identified in the lower-order target area at (1) only infragranular layer (layers 5 and 6), (2) only supragranular layer (layers 1–3), (3) both supragranular and infragranular layers (layers 1–3, and 5 and 6), and (4) only layer 1^51,52^. In the present study, we have identified the inter-areal signal for retrieving the to-be-recalled object, which was directed at the supragranular layer during cue perception and at the infragranular layer during memory retrieval. Taken these anatomical observations into account, our findings suggest that the anatomical basis of functional connectivity in cluster 1 is pattern (3), in which A36 neurons project to both supragranular and infragranular layers of TE, and the signal along the projections from A36 neurons to both layers in TE is not functionally static, but flexibly recruited for cued-recall by switching the target layer, depending on cognitive operations involved.

In the present study, we employed STAγ, a neuronal index of spike-triggered average of the TE γ-power, to capture the impact of the inter-areal signal between spiking activity of A36 neuron and LFP in TE, on inter-laminar signal processing in TE^42^. In other words, STAγ represents the coupling of neuronal activity among three sites, one in A36 (spiking activity) and two in TE (LFPs). Thus, STAγ is different from PAC, which captures the LFP signal coordination between two sites in TE. Also, STAγ is different from coherence between one site in A36 (spiking activity) and on site in TE (LFP). Using these distinct neuronal measurements, we revealed dynamics of inter-areal and inter-laminar signal for successful memory retrieval in cued-recall. Although the results for STAγ and PAC were similar in several points, i.e., the modulation of gamma envelope was observed at 10−20 Hz both in STAγ and PAC during delay period and the laminar pattern of both the metrics were similar, the results were different in the error analysis. Specifically, the inter-laminar (i.e., within-areal) signaling in TE, captured by PAC, was constant between correct and error trials, whereas coupling between “inter-areal interaction between A36 and TE” and “inter-laminar signaling in TE”, captured by STAγ, differed depending on the monkey’s task performance. In addition to PAC, coherence between A36 and TE also did not differ between correct and error trials. Thus, behaviorally relevant mnemonic signals could emerge in the coupling between inter-areal and inter-laminar signal processing, without behaviorally relevant inter-areal or inter-laminar signals themselves. One plausible hypothesis for the neuronal circuit machinery underlying successful memory retrieval is that the inter-laminar signaling in TE is utilized for animals’ behavior, in the context of its interaction with A36.

In the present study, we found that the inter-areal signal between A36 and TE dynamically changed not only the target layer, but also the frequency range of coherent activity between these two areas. Previous studies have demonstrated that oscillatory activities in CA1 of the hippocampus coupled with those in both the medial entorhinal cortex and CA3 in fast and slow γ frequency ranges, respectively^53^. Considering that oscillatory activity in LFP reflects an emergent property of network activity, changes in the processing within the cortical circuit would result in changes in the oscillation frequency. Elucidating the key determinants for the oscillation frequency will be a critical issue for understanding the mechanistic basis underlying brain-wide network operation.

Recently, a simulation-model study has shown that oscillatory synchrony in the γ frequency range induced flexible information routing between distinct cortical regions^54^. The present results provide an experimental basis for the flexible information routing between microcircuits in distinct areas at the resolution of cortical layers and expand our view of flexible communication in the brain-wide network. Moreover, the present results suggest that dynamic laminar routing of inter-areal coordinated activities underlie temporally sequential cognitive behaviors.

## Methods

### Subjects

All the experimental protocols, animal welfare, and procedures for ameliorating suffering were in full compliance with the "Guidelines for Proper Conduct of Animal Experiments" by the Science Council of Japan, with the "Guidelines regarding animal research and animal experimentation" by the University of Tokyo, and with the "Guidelines for the Care and Use of Laboratory Animals" by National Institutes of Health, as well as with the Weatherall report "The use of non-human primates in research". The experimental protocol was approved by the University of Tokyo School of Medicine Animal Care and Use Committee (permission number, MED: P11-098).

Two macaque monkeys (*Macaca mulatta*, both male, weighing 7.4–8.6 kg) were used. MRI-compatible head holders and recording chambers were attached to the skull under aseptic conditions and general anesthesia^33–35,37–40,42,43^. Blood pressure, heart rate, respiratory rate, and oxygen saturation were monitored continuously during the surgery. After the surgery, monkeys were given postsurgical analgesics (acetaminophen, 20 mg kg^−1^ day^−1^, or pranoprofen, 3 mg kg^−1^ day^−1^, per os) for at least 3 days, and postsurgical prophylactic antibiotics (benzylpenicillin, 20,000 unit kg^−1^ day^−1^; ampicillin, 100 mg kg^−1^ day^−1^, intramuscular injection; or enrofloxacin, 5 mg kg^−1^ day^−1^, subcutaneous injection) for 1 week. The monkeys were housed in their own cage with a 13-h light/11-h dark cycle, and experiments were conducted during the light cycle.

To guide placement of the microelectrode, structural images of the brain were acquired using a 4.7 T magnetic resonance imaging (MRI) scanner (Biospec 47/40; Bruker BioSpin, Ettlingen, Germany) and actively decoupled surface receive radiofrequency coil of 50-mm diameter with a volume radiofrequency coil for transmission (Bruker BioSpin). Under propofol anesthesia (5−10 mg kg^−1^ h^−1^, i.v.), we obtained high-resolution structural images of the brain for each monkey using a fast spin-echo sequence (in-plane resolutions, 200 × 200 μm^2^; slice thickness, 1 mm; TE/TR, 60/3000 ms; ETL, 8)^40,55,56^.

### Behavioral task

In each trial, following the presentation of a fixation point for 500 ms, a cue stimulus (1 of the 24 visual stimuli) was presented for 500 ms (Fig. 1c). After a delay period of 2000 ms (or 1000 ms for a subset of datasets in monkey-1), two stimuli were presented, one of which was the paired associate of the cue stimulus, and the other was a distractor. The monkey obtained fruit juice as a reward for correctly touching the paired associate within 1500 ms. Eye movements were monitored with a PC-based CCD camera system^33–36,42^, and if the eye position deviated more than 2.0° (monkey-1) or 1.5° (monkey-2) from the fixation point, the trial was automatically terminated. The performance of the two animals was 80.2 ± 9.0% (mean ± s.d.) in monkey-1 and 75.9 ± 8.3% in monkey-2. The performance rate was significantly higher than the chance level in both monkeys (paired *t*-test, monkey-1, *t* = 14.9, *P* = 5.86 × 10^−12^; monkey-2, *t* = 10.4, *P* = 1.10 × 10^−6^).

### Configuration of electrophysiological recordings

Extracellular recordings were conducted using glass-coated tungsten electrodes [for area 36 (A36)] or linear-array multi-contact electrodes^42,43,48,57^ [U-probe, Plexon Inc, TX, USA; for area TE (TE)] containing 16 recording channels (impedance, 0.3‒0.5 MΩ at 1 kHz) with an intercontact spacing of 150 µm (Fig. 2a).

Neuronal signals were recorded using a Plexon MAP system (Plexon Inc, TX, USA). Each signal was separated into two signals with different band-pass analog filters, higher frequency spiking activities (250 Hz–8 kHz), and lower frequency field potentials (LFPs; 3–88 Hz). These signals were stored in a PC with a sampling rate of 40 kHz for spiking signals and 1 or 20 kHz for field signals. Field signals sampled at 20 kHz were then downsampled to 1 kHz. LFP data were corrected for the possible phase shifts that could be induced by the filters in the system^16^.

At the end of each recording session, the anteroposterior (AP) and lateromedial (LM) coordinates of the electrode track were measured by X-ray imaging^33–40,42,43,55^, and the dorsoventral (DV) coordinates were measured by manipulator readings. In 8 penetrations (6 penetrations in monkey-1 and 2 penetrations in monkey-2), electrolytic lesions were made along the electrode track, with a spacing of 1.5 or 2.0 mm, by passage of a direct current (5–10 μA for 15–20 s)^40,42,43,55,56^. The lesion marks were identified by histological examinations after all recordings had been completed in each monkey.

### Histological estimation of the recording sites

Histological analysis was performed using standard protocols^40,42,43,55,56^. Briefly, coronal 40-μm cryostat sections were collected in four series, and one series of sections was subjected to Nissl staining. The border between A36 and TE or area 35 was cytoarchitectonically determined according to the criteria described in previous studies^58,59^. Photomicrograph images of the sections were obtained using a Keyence BZ-9000 microscope system (Keyence, Osaka, Japan).

Photomicrograph images of the histological sections were aligned and reconstructed into a volume by referring to fiducial pinholes, which had been punctured vertically into the sectioning plane using a 26-gauge needle during cryosectioning. To estimate the positions of the recording site in the histological sections, each recording site determined by X-ray-based coordinates was manually rigid-transformed into histological space by means of metal deposit positions, which were measured by both X-ray imaging and histological sections, as described in Koyano et al.^40,55^. Thirty-three metal deposits (14 marks for monkey-1 and 19 marks for monkey-2) at the IT cortex were used to minimize errors arising from global tissue distortion. Shrinkage rates of histological sections (5.9–9.1%) were estimated for each monkey by comparing the distance between 3D coordinates of the metal deposits of the electrodes on the histological sections and 3D coordinates of the electrodes measured during recording sessions^40,55^. The 3D coordinates of the electrodes during recording sessions were determined by X-ray images [anterior-posterior (AP) and lateral-medial (LM)] with manipulator readings (dorsal-ventral, DV). The metal deposits had been created by passing a current to elgiloy electrodes that were used for single unit mapping. After correcting for tissue shrinkage, the localization accuracy of the recording sites was calculated as the mean distance between the position of the electrolytic lesion marks, estimated from the X-ray with manipulator readings and the position of the lesion marks actually found on the histological sections. The distribution of the localization accuracy of the electrolytic lesion marks was −0.01 ± 0.27 mm (AP), 0.09 ± 0.16 mm (LM), and −0.01 ± 0.13 mm (DV). The angles of penetrations were estimated by overlying the electrode paths on the corresponding histological sections. Penetrations in which the estimated angle between the electrode path and the direction perpendicular to the cortex exceeded 20 degrees were not included in the analysis^43^.

### Neuronal database

We recorded neuronal activity of a total of 96 neurons in A36 with LFPs in TE. Then, we obtained 93 datasets (simultaneously recorded spiking activity in A36 and LFP in TE), in which A36 neurons showed significant stimulus selectivity (*P* *<* 0.01) both during the cue period (70−570 ms from cue onset) and the delay period (200−2000 ms from cue offset, or 200−1000 ms for a subset of data in monkey-1) using one-way ANOVA. Among these datasets, we selected the datasets according to the following criteria: an A36 neuron has a cue stimulus that produced (1) the strongest delay activity and (2) activity ranked in the top-four among the responses evoked from 24 stimuli during the cue period. We defined the optimal trial as all trials in which the above-determined cue stimulus was presented. The data were included in further analyses only when the optimal stimulus could be determined for the A36 neuron. Accordingly, a total of 56 datasets was used for the subsequent analyses. The mean number of optimal trials was 79.4 (±28.8, s.d.) trials.

### Estimation of cortical layers

To estimate the cortical depth of the linear array electrode in TE, we conducted a current source density (CSD) analysis^42–44,48,57,60^ of the stimulus-evoked LFPs by the same procedure used in Takeuchi et al.^43^ and Takeda et al.^42^. Briefly, we defined the channels showing the earliest current sink in the CSD profiles as the zero point of the CSD profiles. We then aligned the Gr channel at the center of the histological granular layer to locate each electrode channel on the histological section. Note that channels in SG or IG did not include the neighboring (<0.3 mm) channels on either side of the channel that exhibited the earliest sink^42,43^. In terms of cortical localization of A36 neurons, we could not use CSD, because single channel tungsten electrodes were used for A36. Thus, we estimated the cortical location of A36 neurons by aligning coordinates of the recording sites on the histological sections via the coordinates of the lesion marks in the area. Note that we classified only whether a neuron was located above or below the center of the granular layer. The numbers of neurons located above (*n* = 33) and below (*n* = 23) the center of the granular layer were not statistically different (*χ*^2^ = 1.79, *P* = 0.229). We normalized the relative cortical location of the A36 neurons by the location of the pia mater and white/gray matter boundary. Differences in normalized cortical depth of A36 neurons across clusters were marginally significant (*χ*^2^ = 5.81, *P* = 0.0548, Kruskal−Wallis test).

### Response properties of A36 neurons

To examine the signal contents conveyed by A36 neurons, we defined the cue-holding index (CHI), the pair-recall index (PRI), and the pair-coding index (PCI), which were used in previous studies^33,34^: CHI = (*R*_**C**|**D**_ − *R*_**C**|**C***p*_*R*_**C***p*|**D**_)/[(1 − *R*_**C**|**C***p*_^2^) (1 − *R*_**C***p*|**D**_^2^)]^1/2^, PRI = (*R*_**C***p*|**D**_ − *R*_**C**|**C***p*_*R*_**C**|**D**_)/[(1 − *R*_**C**|**C***p*_^2^) (1 − *R*_**C**|**D**_^2^)]^1/2^, and PCI = *R*_**C**|**C***p*_, where *R*_**A**|**B**_ denotes a correlation coefficient between **A** and **B**. The vectors of **C** and **C**_*p*_ denote the cue-period responses for the set of 24 cue stimuli, **C**: [*c*_1_, …, *c*_24_] and **C**_*p*_: [*c*_*p*(1)_, …, *c*_*p*(24)_], where the *i*-th and *p*(*i*)-th stimuli belong to a pair. The vector of **D** denotes the delay-period responses for the 24 cue stimuli, **D**: [*d*_1_, …, *d*_24_]. For CHI and PRI, we employed partial correlation coefficients because a neuron in the IT cortex tends to encode both paired stimuli^34^, so that the correlation between **C** and **C**_*p*_ must be removed in the analysis of **D**. In Figure 3b−c and Supplementary Figure 4, we selected datasets showing a significant PRI/PCI value at a threshold of *P* < 0.05.

### Inter-areal coherence

Coherence between A36 spiking activities and TE LFPs^42^ was calculated using Chronux toolbox (http://chonux.org/) for Matlab (MathWorks, MA, USA). To assess the statistical significance, a coherence spectrum during the cue and delay periods was calculated using both the original and trial-shifted spike trains. The trial-shifted control of coherence is the coherence calculated by shifting the trial order of spike trains (a shift of one trial in time). Thus, the spike train of one trial of one A36 neuron corresponds with the TE LFP in a different trial. This procedure, often called as a shift predictor, is widely used for removing spurious covariation (e.g., stimulus-locked responses) between simultaneously recorded neuronal signals^61^. The trial-shifted coherence spectrum was then subtracted from the original coherence spectrum to construct the shift-predictor-subtracted-coherence. For each dataset, coherence for each TE channel was sorted along the distance from Gr that was identified by CSD analysis.

### Laminar transition of inter-areal coherence

To analyze the transition in laminar specificity of inter-areal coherence from the cue period to the delay period, we performed probabilistic principal component analysis (PCA)^62^ and cluster analysis^40,42^ as follows (*n* = 56 datasets; Fig. 3). First, we conducted PCA of 52-dimensional vectors that were composed of mean coherence values [coherence profile: 2 periods (cue and delay); 2 frequency ranges (10–25 and 25–40 Hz); 13 channels]. We then classified the coherence profiles into subsets (clusters) according to k-means clustering, which was applied to the PC space (the three-dimensional subspace for PCs 1–3 is shown in Fig. 3c). For details about determination of number of clusters in the cluster analysis, see the section titled Cluster analysis and choice of number of clusters. Because the vectors included the coherence values during both the cue and delay periods, the cluster analysis used in this study allowed extraction of the transition pattern of coherence from the cue period to the delay period in each cluster. A five-way ANOVA for clusters (1–3), monkeys, layers (IG and SG), periods (cue and delay), and frequencies (10–25 and 25–40 Hz) showed no significant main effect of monkeys (*F* = 0.00470, *P* = 0.946; see also Supplementary Fig. 10 for reproducibility of clustering for respective monkeys).

### Cluster analysis and choice of number of clusters

We tested the optimal number of clusters by using five methods. We also tested the cluster stability using Jaccard coefficients. Details are as follows.

Firstly, we tested the optimal number of clusters to be divided by Jain-Dubes method^63^ (Supplementary Fig. 6a). In the Jain-Dubes method, the optimal number of clusters is estimated by

$$p\left( m \right) = \frac{1}{m}\mathop {\sum }\limits_{i = 1}^m \max _{1 \le j \le m}\left\{ {\frac{{\eta _i + \eta _j}}{{\varepsilon _{ij}}}} \right\}$$

where

$$\eta _j = \frac{1}{{n_j}}\mathop {\sum }\limits_{i = 1}^{n_j} D\left( {{\mathbf{F}}_i^{(j)},\;{\mathbf{c}}_j} \right),\;\varepsilon _{ij} = D({\mathbf{c}}_i,\;{\mathbf{c}}_j)$$

${\mathbf{F}}_i^{(j)}$is an *i*th vector in the cluster *C*_*j*_, **c**_*j*_ is a centroid of *C*_*j*_, and *D* is the distance between two data points. The optimal number of clusters is *m* that minimized *p*(*m*). Our results showed that *p*(*m*) was minimum when the number of clusters was three.

Secondly, we employed the upper-tail methods^64^, by which the optimal number of clusters is the first *j* that satisfies

$$\alpha _{j + 1} \,> \, \bar \alpha + ks_\alpha$$

where $\alpha _{j + 1}$ represents the value of the criterion in the stage *j*+1, *k* is the standard deviate, and $\bar \alpha$ and *s*_*α*_ are the mean and unbiased standard deviation of the *α* distribution, respectively. In the present data, the optimal number of clusters was three when the standard deviate *k* was set as three. This deviate value fell within the range of two to four that was used in Mojena^64^. Note that the upper-tail method can be used for hierarchical clustering.

Thirdly, we employed gap criterion^65^ (Supplementary Fig. 6b), under which the optimal number of clusters occurs at the solution with the largest gap value. The gap value is defined as

$${\mathrm{Gap}}_n\left( k \right) = E_n^ \ast \left\{ {\log \left( {W_k} \right)} \right\} - \log \left( {W_k} \right)$$

where *n* is the sample size, *k* is the number of clusters being evaluated, and *W*_*k*_ is the pooled within-cluster dispersion measurement, calculated by:

$$W_k = \mathop {\sum }\limits_{r = 1}^k \frac{1}{{2n_r}}D_r$$

where *n*_*r*_ is the number of data points in cluster *r*, and *D*_*r*_ is the sum of the pairwise distances for all points in cluster *r*. The expected value $E_n^ \ast \left\{ {\log \left( {W_k} \right)} \right\}$ is determined by Monte Carlo sampling from a reference distribution, and $\log \left( {W_k} \right)$ is computed from the sample data. The optimal number of cluster (*k*) is determined as the minimum *k* that satisfies ${\mathrm{Gap}}\left( k \right) > {\mathrm{Gap}}\left( {k + 1} \right) - {\mathrm{standard}}\;{\mathrm{error}}$. Gap statistics depicted that the optimal number of clusters was three.

Fourthly, we tested the optimal number of clusters by Calinski–Harabasz criterion^66^, also known as the variance ratio criterion (VRC) (Supplementary Fig. 6c). The Calinski–Harabasz index is defined as

$${\mathrm{VRC}}_k = \frac{{{\mathrm{SS}}_{\mathrm B}}}{{{\mathrm{SS}}_{\mathrm W}}} \times \frac{{\left( {N - k} \right)}}{{\left( {k - 1} \right)}}$$

where SS_B_ is the overall between-cluster variance, SS_W_ is the overall within-cluster variance, *k* is the number of clusters, and *N* is the number of observations. The optimal number of clusters is the solution with the highest Calinski–Harabasz index. The Calinski–Harabasz criterion depicted that the optimal number of clusters was three.

Finally, we tested the optimal number of clusters using the silhouette criterion^67^ (Supplementary Fig. 6d). The silhouette value for each data point is a measure of how similar that point is to points in its own cluster, when compared to points in other clusters. The silhouette value for the *i*th point, *S*_*i*_, is defined as

$$S_i = \frac{{\left( {b_i - a_i} \right)}}{{{\mathrm{max}}\left( {a_i,b_i} \right)}}$$

where $a_i$ is the average distance from the *i*th point to other points in the same cluster as *i*, and *b*_*i*_ is the minimum average distance from the *i*th point to points in a different cluster, minimized over clusters. The silhouette value ranges from −1 to +1, with a higher value indicating that the point is well-matched to its own clusters. The number of clusters that maximize average *S*_*i*_ over all data points is considered to be optimal. The silhouette criterion depicted that the optimal number of clusters was three. In Supplementary Figure 6e, we present the resulting silhouette plot. Most data depicted positive silhouette values, suggesting that data in each cluster are tightly grouped, and that the overall data are appropriately clustered.

All of these statistics indicated that the optimal number of clusters to be divided was three. We additionally tested whether the divided three clusters were statistically stable. For this purpose, we employed a recently developed stability test^68^ that uses Jaccard coefficient (Supplementary Fig. 6f). The Jaccard coefficient for clusters 1, 2, and 3 was 0.785, 0.756, and 0.652, respectively, all above 0.6, which constitutes the criteria of stability. To test the statistical significance, we then compared these coefficient values with the distribution of coefficient values generated by shuffling data. The Jaccard coefficient for clusters 1, 2, and 3 fell in the top 0.01% of the distribution. These results suggest that clustering into clusters 1, 2, and 3 in the present study is significantly stable.

### Impact of inter-areal coherence on inter-laminar processing

Several previous studies took the γ-power as an index of local neural processing^5,45^ and investigated the relationship between spiking activity and γ activity^69^. In each dataset, the γ-power of the LFP (40−90 Hz) during the cue and delay periods was calculated for each TE channel. We then examined the contribution of inter-areal coherence to the coupling of A36 spikes with TE γ-power during the two distinct periods, as follows. The instantaneous amplitude and phase of the LFP were extracted by convolving the raw LFP with a complex Morlet wavelet transform^9^ (5-Hz resolution). The resultant time-varying amplitude of γ LFP was used as the γ-power envelope of the LFP^12,42,57^. The spike-triggered average of the γ-power envelope (STAγ) at the channel showing maximal γ-power (γ-power-channel) was then calculated between A36 spikes and TE LFPs, as follows. First, the instantaneous phase value of the TE LFP at the frequency of the maximum inter-areal coherence between 10 and 40 Hz (φ_max_) was defined as the relative phase of each A36 spike. Then, spikes firing in the phase ranging within ±1/4 *π* of φ_max_ were extracted as coherent spikes, and those firing in the opposite quadrant were extracted as non-coherent spikes. The STAγ was subsequently calculated for coherent and non-coherent spikes separately, as the average of the gamma envelopes within ±100 ms from each spike. The same calculations were performed 1000 times following trial-shuffling between A36 spiking activities and the γ-power envelope of the TE LFPs to compute the shuffle-predictor STAγ. The shuffle-predictor was then subtracted from the original STAγ to construct the shuffle-predictor-subtracted STAγ, which was normalized with the SD of the shuffle-predictor for each time lag. This procedure resulted in the normalized *z*-score-transformed STAγ. The spectral power of the resultant z-score-transformed STAγ was then calculated to evaluate its temporal periodicity, and the average power in the frequency range of 13−23 Hz (cue period) and 5−18 Hz (delay period) (STAγ power) was compared between correct and error trials. The TE LFP phases at the timing of A36 spikes showed non-uniform probability distribution (i.e., phase-locking), and coherent spikes showed a more concentrated probability distribution of the LFP phases than did non-coherent spikes. Thus, greater STAγ power with coherent spikes rather than with non-coherent spikes might contain contributions of intra-areal phase-amplitude coupling (PAC) between γ activity and the phase at the frequency of coherence with A36 spikes. Thus, we calculated STAγ by equalizing the number of spikes and the phase concentration of spikes between coherent and non-coherent spikes, by randomly deleting spikes in each trial and jittering non-coherent spikes to equalize the overall phase concentration of spikes between coherent and non-coherent spikes. To reduce any possible contribution of trial number differences between correct and error trials on STAγ power (Fig. 4), the spiking activity in A36 (Supplementary Fig. 16a), the LFP γ-power in TE (Supplementary Fig. 16b, c), and the coherence between A36 and TE (Supplementary Fig. 16d, e), we also equalized the number of trials between correct and error trials by randomly removing correct trials.

We also calculated PAC within and between layers in TE as follows (Supplementary Fig. 17). To quantify PAC in a single scalar, we used an approach known as the modulation index (MI)^57,70^. The statistical significance of the MI values was assessed as follows. We randomly shifted the phase time series, and computed MI using this shifted signal. We repeated this procedure 100 times, resulting in a distribution of MI values. We subsequently normalized MI using the mean and standard deviation of the MI distribution. For comparison with the STAγ, we averaged the normalized MI between low-frequency LFP (13−23 Hz for cue period and 5−18 Hz for delay period) at a phase channel and high-frequency LFP (40−90 Hz) at an amplitude channel.

### Statistical analysis

Data analyses were conducted using MATLAB (Mathworks) and R software. Statistical tests were two-sided. We corrected *P*-values for multiple comparisons by Bonferroni’s method when necessary. Unless otherwise stated, center values and error bars in the figures depict the mean and the standard errors of the mean (s.e.m.), respectively. The variance was similar between the groups that were being statistically compared, but in Supplementary Figure 13, we used median and lower/upper quartiles (non-parametric analysis) due to skewed distribution of the data. Blinding was not performed in the analyses. The experiments were not randomized to determine how samples/animals were allocated to experimental groups. No statistical methods were used to predetermine the sample size. However, our sample size for numbers of animals and behavioral experiments were similar to those reported in previous publications^34,37,40,43^.

## Electronic supplementary material

Supplementary Information

## Data Availability

The data that support the findings of this study are available from the corresponding author upon reasonable request.
