# Supplementary material for: Dynamic laminar rerouting of inter-areal mnemonic signal by cognitive operations in primate temporal cortex
Source: Nat Commun. 2018 Nov 6;9:4629. doi: 10.1038/s41467-018-07007-1 (PMC6219507; doi:10.1038/s41467-018-07007-1)
Supplement: Supplementary file 1 — Supplementary Information [file 41467_2018_7007_MOESM1_ESM.pdf]

Supplementary Information

Title: Dynamic laminar rerouting of inter-areal mnemonic signal by cognitive operations in primate temporal cortex

Authors: Takeda et al.

Representative coherence

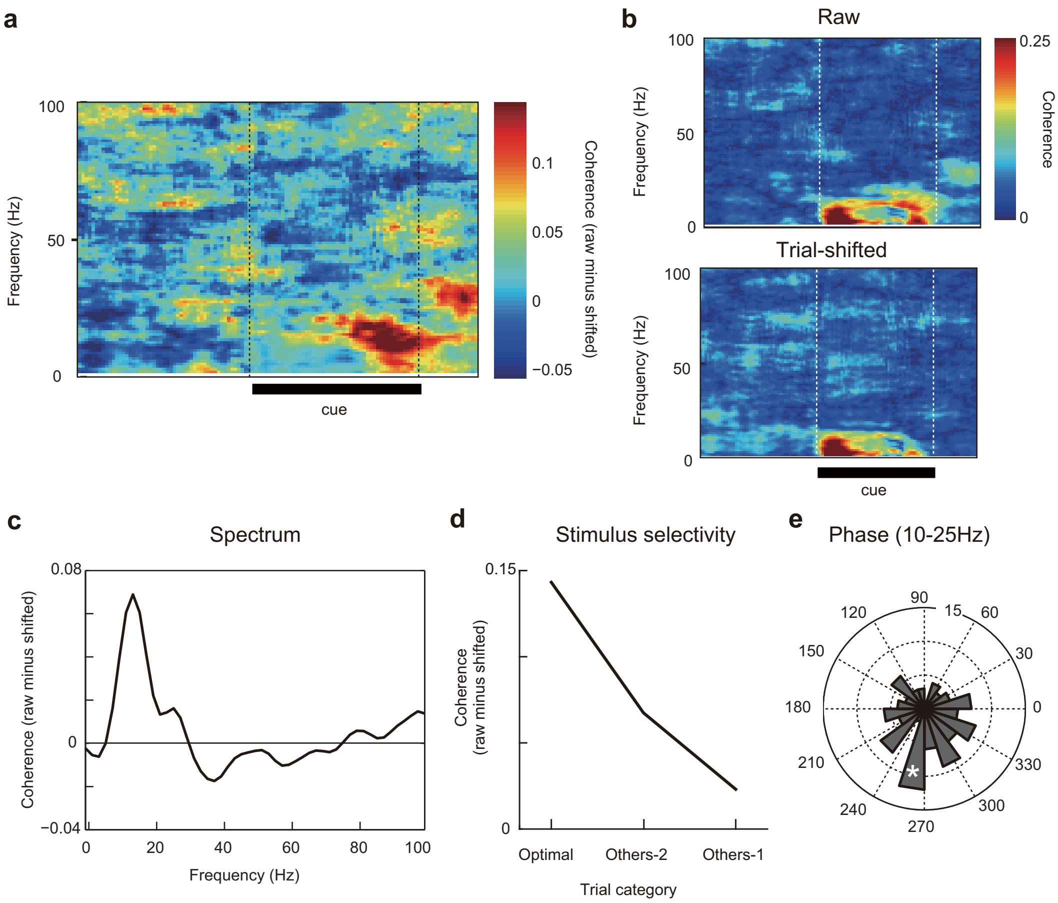

Supplementary Figure 1

Supplementary Figure 1 | A representative "A36 spike-TE LFP" coherence during the cue period. (**a**) Temporal dynamics of coherence (coherogram). Subtraction of trial-shifted control from raw coherence is shown. During the cue period, coherence was elevated at frequencies < 25 Hz. (**b**) Raw coherogram and trial-shifted coherogram. (**c**) Power spectrum of coherence during the cue period. (**d**) Stimulus selectivity of coherence. Optimal, the optimal trial. Others-2, trials in which the cue stimuli elicited the 13th to 18th largest spiking responses. Others-1, trials in which the cue stimuli elicited the 19th to 24th largest spiking responses. (**e**) Distribution of mean angular phase at 10–25 Hz in each trial. The phase angle of 0 degree was set at the trough of the TE LFP, and negative phase values (180–360 degree) indicate that spikes of the A36 neuron tended to fire at the falling phase of the TE LFP. *, Rayleigh test; $z = 5.84$, $P = 0.00275$.

Population coherence

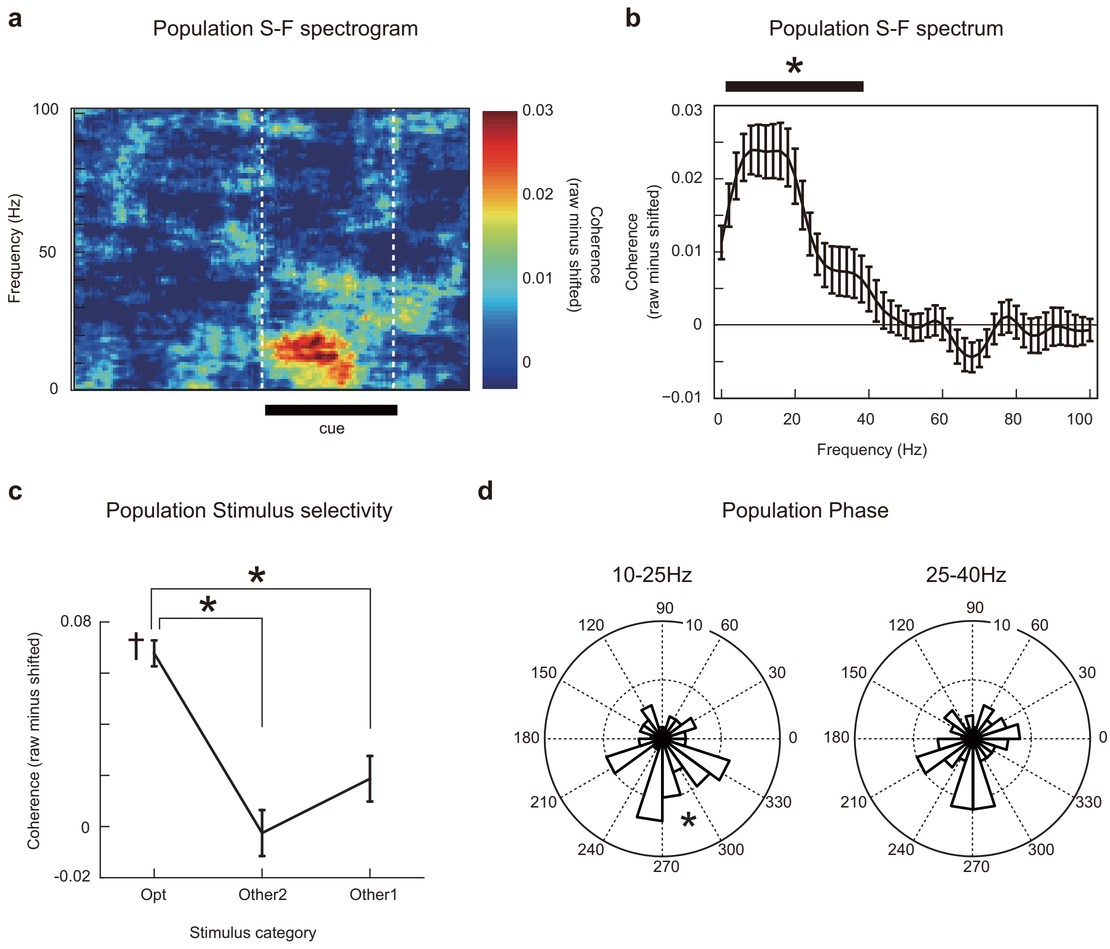

Supplementary Figure 2

Supplementary Figure 2 | Population inter-areal coherence during the cue period. (**a**) Temporal dynamics of coherence (coherogram; n = 56). Subtraction of the trial-shifted control from raw coherence is shown. (**b**) Power spectrum of coherence (mean ± s.e.m.) during the cue period. Peak coherence was observed at around 10−20 Hz. *, $P < 0.01$; paired t-test, corrected for multiple comparisons. Note that coherence was significant at frequencies < 40 Hz. (**c**) Stimulus selectivity of coherence (mean ± s.e.m.). Opt, the optimal trial. Others-2, trials in which the cue stimuli elicited the 13th to 18th largest spiking responses. Others-1, trials in which the cue stimuli elicited the 19th to 24th largest spiking responses. *, Tukey–Kramer test ($P < 0.01$) after one-way ANOVA ($F = 18.7$, $P = 8.21 \times 10^{-8}$). †, comparison with zero (paired t-test, $P < 0.001$, corrected for multiple comparisons). (**d**) Distribution of mean angular phase of coherence at 10–25 Hz (left) and 25–40 Hz (right). The phase angle of 0 degree was set at the trough of the TE LFP, and negative phase values (180–360 degree) indicate that spikes of the A36 neuron tended to fire at the falling phase of the TE LFP. 10–25 Hz: $z = 3.69$, $P = 0.0244$; 25–40 Hz: $z = 1.79$, $P = 0.167$; Rayleigh test.

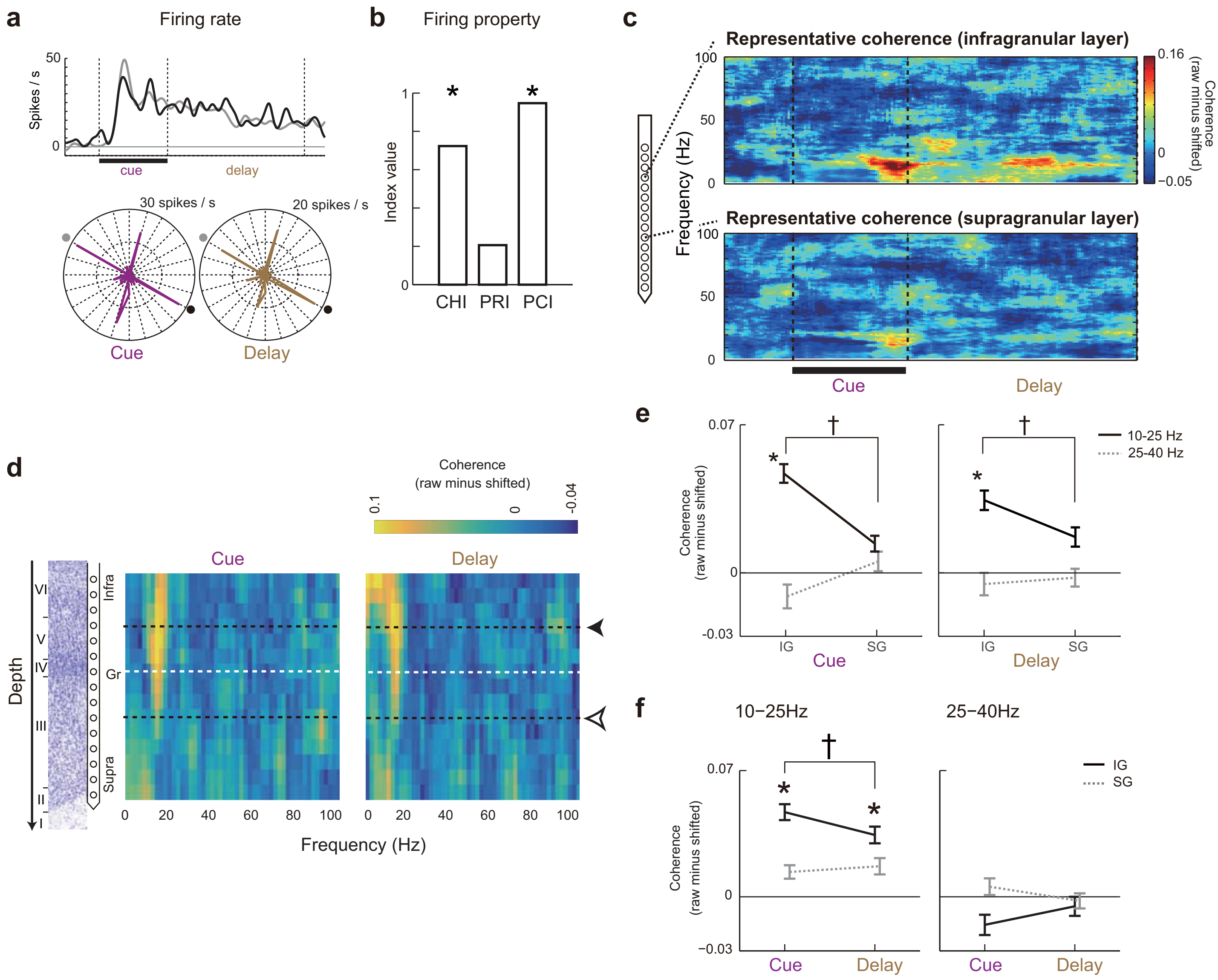

Supplementary Figure 3

Supplementary Figure 3 | Representative data showing non-transition in layer specificity of the inter-areal signal. Figure configuration is the same as Fig. 2b–g. The A36 neuron responded to both the optimal cue stimulus and its paired associate (**a**, **b**). During both the cue and delay period, the inter-areal coherence was larger in the infragranular layer than in the supragranular layer of TE at 10–25 Hz (**c**–**f**). CHI, cue-holding index; PRI, pair-recall index; PCI, pair-coding index; IG, infragranular layer; SG, supragranular layer.

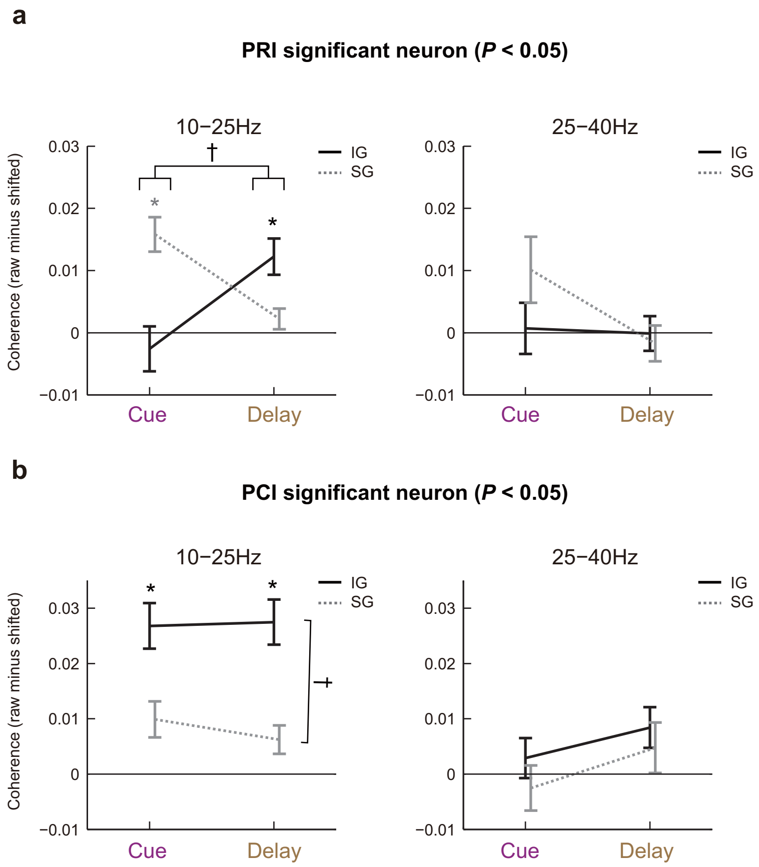

Supplementary Figure 4

Supplementary Figure 4 | Laminar pattern of coherence with neurons showing significant signal contents (PRI/PCI). *, $P < 0.05$; comparison with zero by paired *t*-test corrected for multiple comparisons. †, $P < 0.05$; interaction in ANOVA. PRI, pair-recall index; PCI, pair-coding index; IG, infragranular layer; SG, supragranular layer. Error bars, mean ± s.e.m.

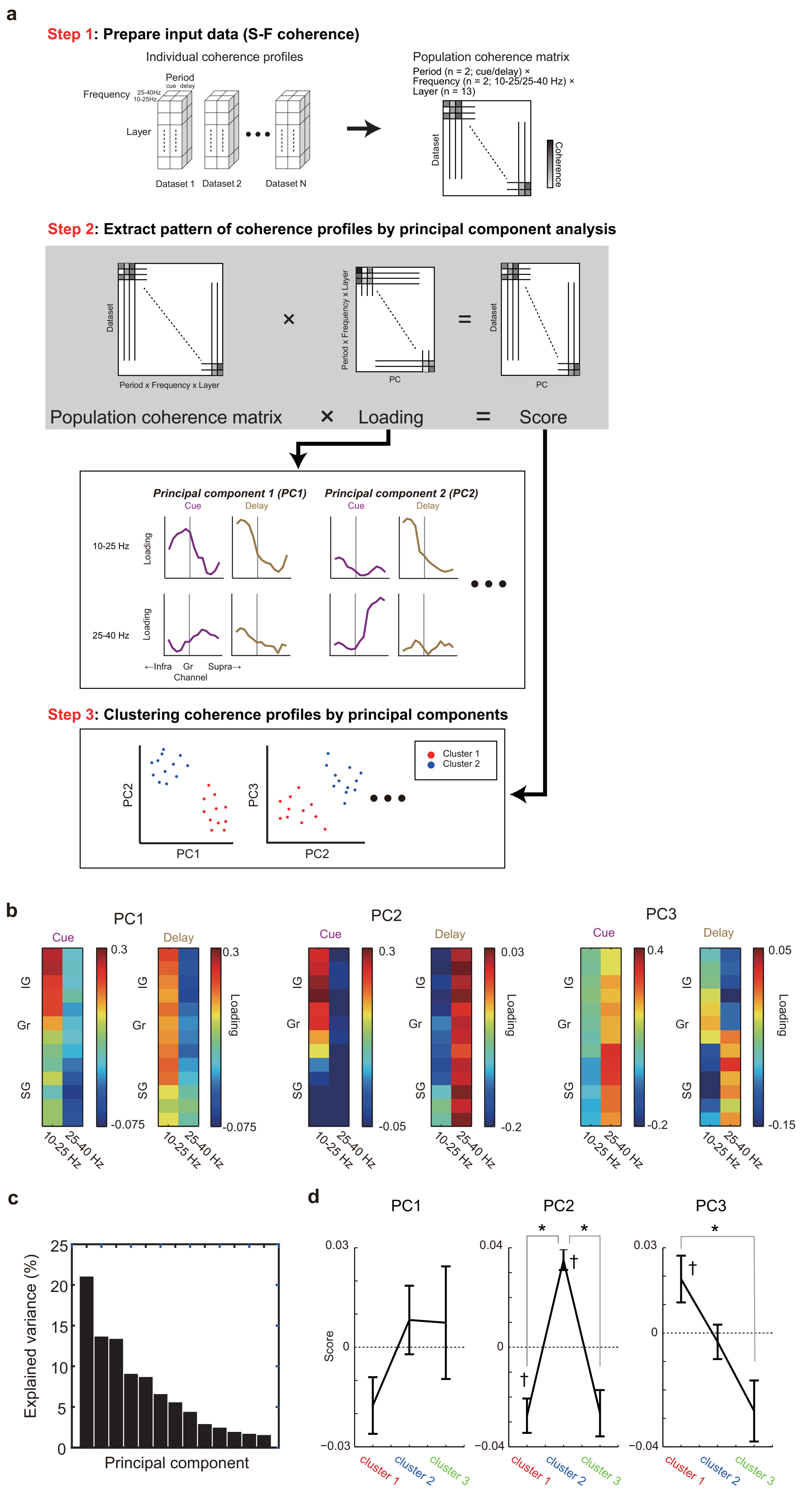

Supplementary Figure 5

Supplementary Figure 5 | Principal component analysis (PCA) for extracting the laminar pattern of coherence. (**a**) Diagram of extraction of the laminar pattern of coherence. Mean coherence values at 10–25 and 25–40 Hz during the cue and delay periods (coherence profiles) were concatenated to create a 52-dimensional vector [2 (periods) × 2 (frequencies) × 13 (layers)] for each dataset. PCA was then conducted to the vectors of all datasets for extracting the pattern of coherence profiles. Principal component (PC) scores are equal to the linear combination of population coherence profiles (mean centered) with PC loadings as coefficients. Finally, each dataset was grouped into clusters according to the cluster analysis of the PCs of each dataset. (**b**) The first three PC loading profiles for the cue and delay periods. The PC1 differentiated coherence between IG and SG during both the cue and delay periods at 10–25 Hz in the same direction. The PC2 differentiated coherence between IG and SG during the cue period at 10–25 Hz. The PC3 differentiated coherence between IG and SG during the cue (25–40 Hz) and delay periods (10–25 Hz) in the opposite direction. (**c**) Variance explained by PCs. (**d**) Comparison of PCs (± s.e.m.) across clusters. In both PC2 and PC3, scores of the PCs were significantly different across clusters ($F = 36.56$, $P < 0.0001$ for PC2; $F = 7.24$, $P = 0.0017$ for PC3). *, Tukey–Kramer test ($P < 0.05$) after one-way ANOVA. †, comparison with zero (paired *t*-test, $P < 0.05$, corrected for multiple comparisons).

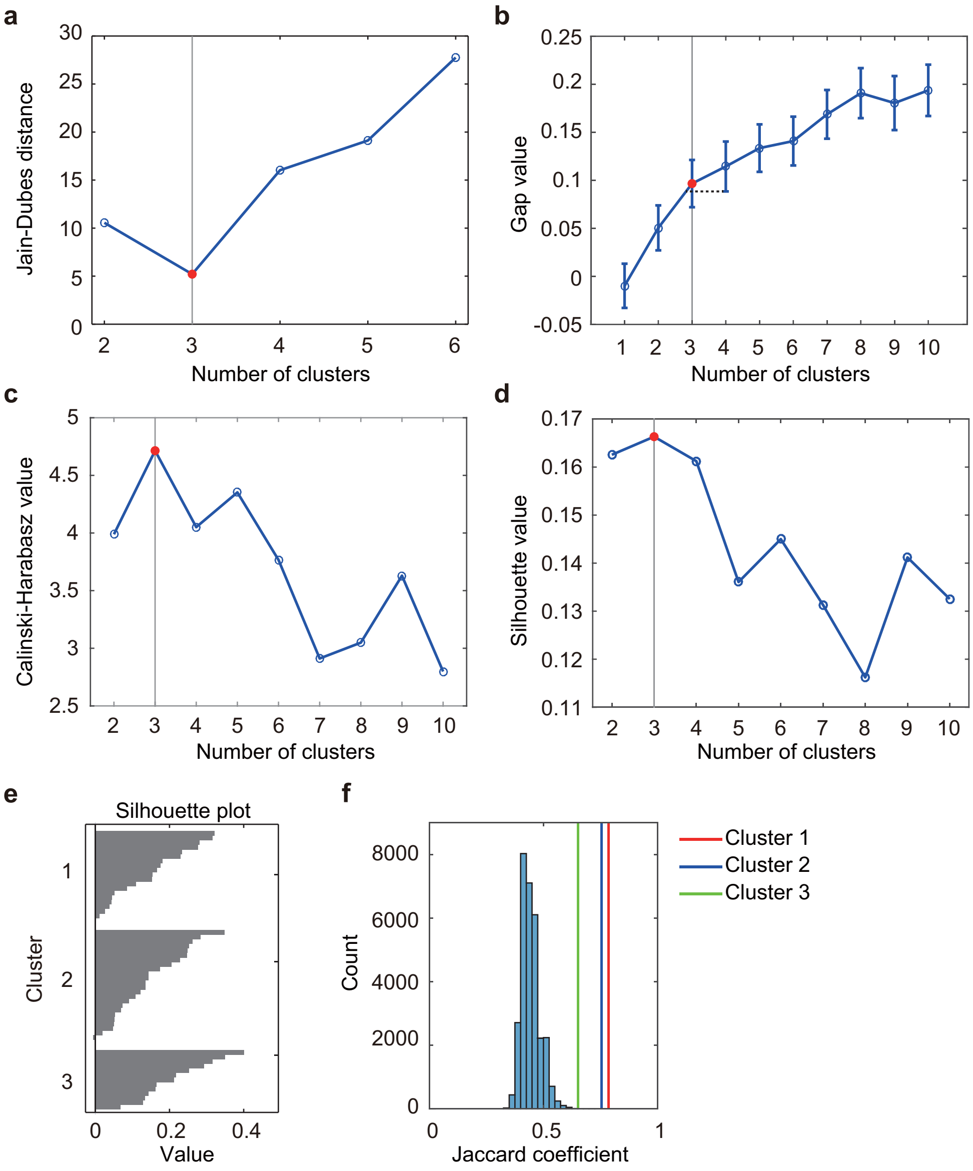

Supplementary Figure 6

Supplementary Figure 6 | Estimation of optimal number of clusters and examination of cluster stability. (**a**) Jain-Dubes distances as a function of number of clusters. (**b**) Gap values as a function of number of clusters. Note that the gap value at three clusters firstly exceeds the gap value at four clusters minus standard error (dotted line). (**c**) Calinski-Harabasz distances as a function of number of clusters. (**d**) Silhouette values as a function of number of clusters. Note that all procedures in (**a**–**d**) depict that the optimal number of clusters to be divided is three. (**e**) Silhouette plot at three clusters. Most data depict positive silhouette values, suggesting that data in each cluster are tightly grouped, and that the overall data is appropriately clustered. (**f**) The Jaccard coefficient for clusters 1 (red, 0.785), 2 (blue, 0.756), and 3 (0.652). Blue histogram depicts the distribution of Jaccard coefficient values generated by data shuffling (n = 10,000 for each cluster). The Jaccard coefficients for clusters 1, 2, and 3 were in the top 0.01 % of the distribution, suggesting that clustering into clusters 1, 2, and 3 is significantly stable. See "Cluster analysis and choice of number of clusters" section in the Methods for details.

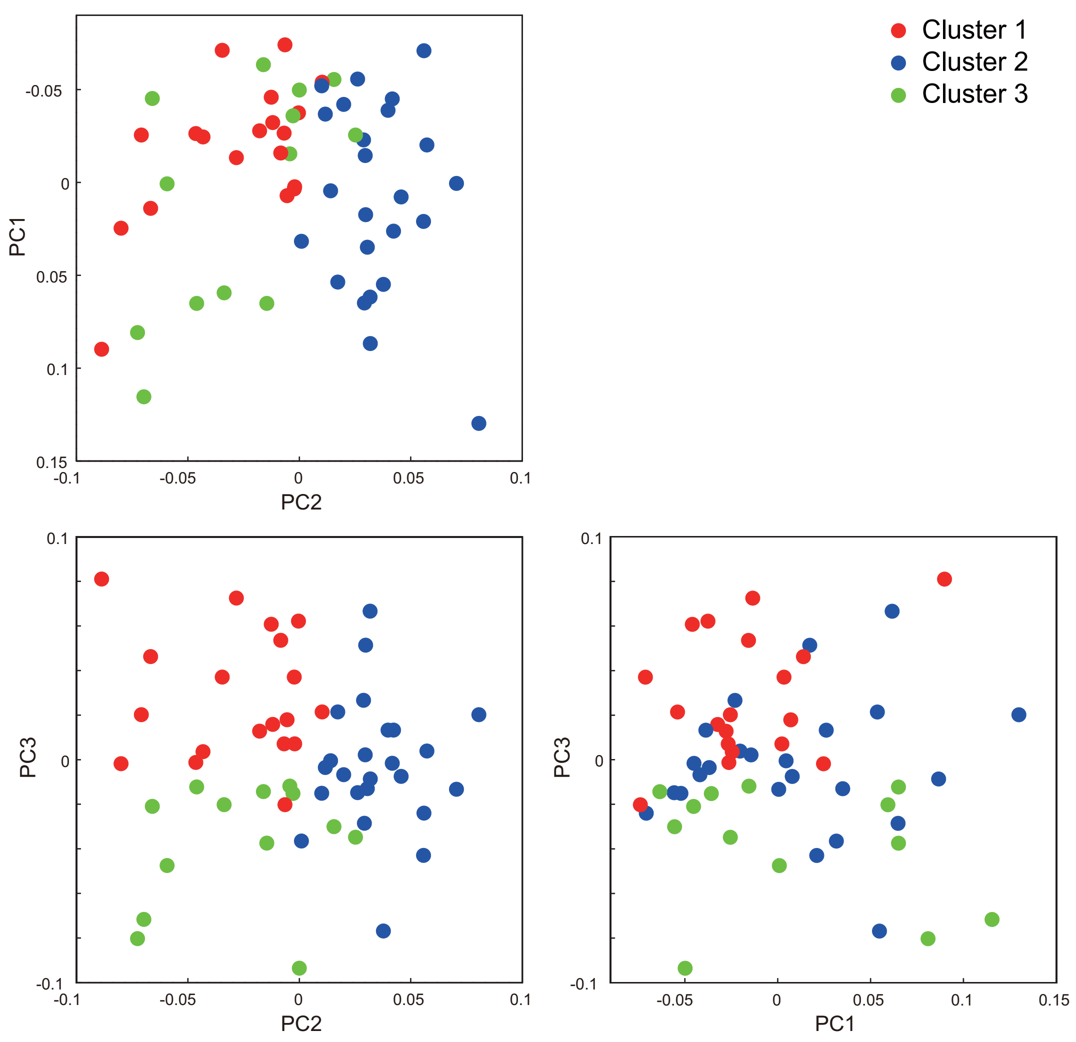

Supplementary Figure 7

Supplementary Figure 7 | Two-dimensional plots for principal component (PC) values of individual datasets. Each dataset is color-coded for the clusters determined by k-means clustering (red = cluster 1, blue = cluster 2, green = cluster 3).

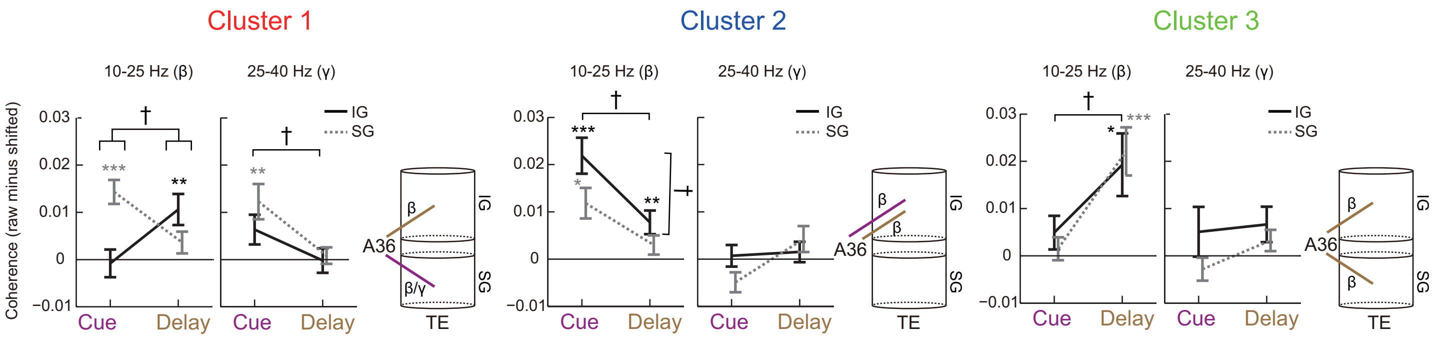

Supplementary Figure 8

Supplementary Figure 8 | Comparison of layer specificity of population coherence between cue and delay periods. The population coherence shown in Figure 3 was directly compared between the cue and delay periods in each cluster. In cluster 1, the two-way ANOVA for period effect (cue/delay) and layer effect [infragranular (IG)/supragranular (SG)] revealed a significant interaction between periods and layers ($F = 25.22$, $P < 0.0001$) at the 10–25 Hz frequency range, and the synchrony at both IG and SG was different between cue and delay periods ($P = 0.0081$ at IG and $P < 0.0001$ at SG). We also found a significant period effect ($F = 7.19$, $P = 0.015$) at 25–40 Hz. In cluster 2, both the period effect ($F = 29.34$, $P < 0.0001$) and the layer effect ($F = 10.12$, $P = 0.0042$) were significant at 10–25 Hz. In cluster 3, we found a significant period effect at 10–25 Hz ($F = 18.89$, $P = 0.001$). These results suggest that there is a significant change in the lower-frequency synchrony during cue and delay periods. *, $P < 0.05$; **, $P < 0.01$; and ***, $P < 0.001$; comparison with zero by paired *t*-test, corrected for multiple comparisons. †, $P < 0.05$; ANOVA. Error bars, mean ± s.e.m.

Individual S-F coherence profile

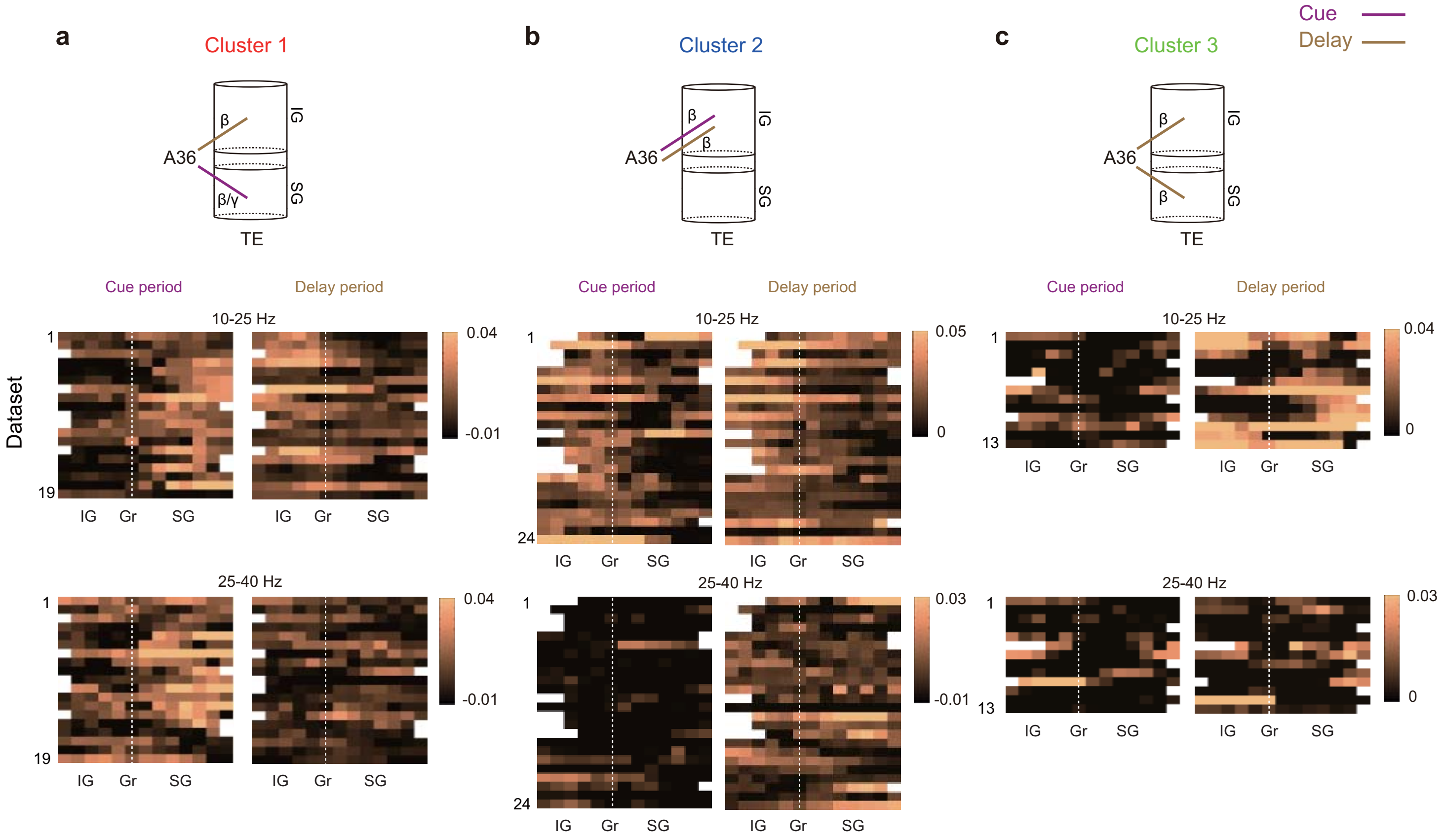

Supplementary Figure 9

Supplementary Figure 9 | Individual depth profiles of inter-areal coherence for cluster 1 (a), cluster 2 (b), and cluster 3 (c). The amplitude of shift-subtracted coherence values is color-coded. IG, infragranular layer; SG, supragranular layer.

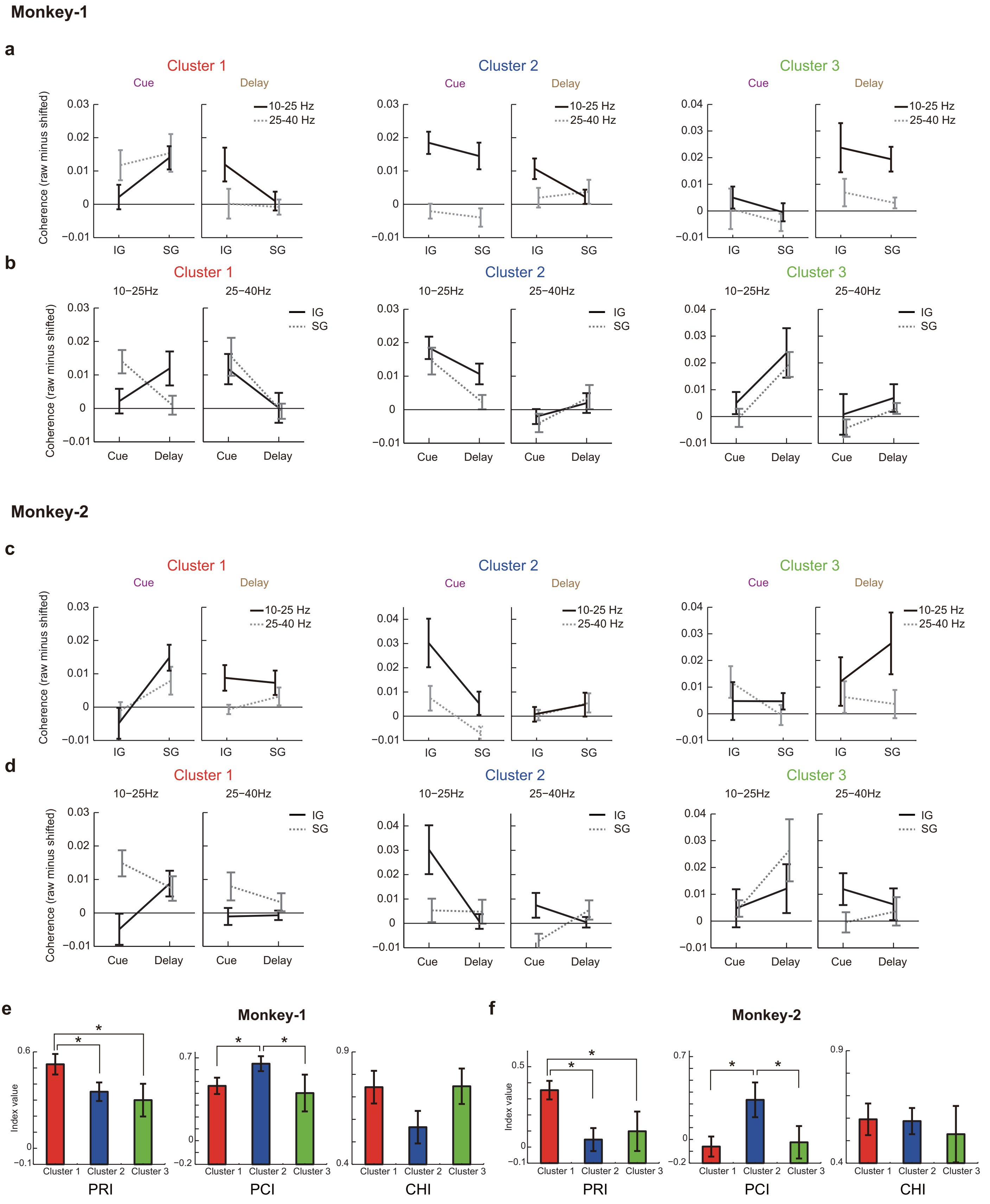

Supplementary Figure 10

Supplementary Figure 10 | Reproducibility of layer specificity of coherence during the cue and delay periods between monkeys. (**a**–**d**) Laminar dynamics of coherence (mean ± s.e.m.) at 10–25 (black) and 25–40 (gray) Hz from the cue (left) to delay (right) periods (**a** and **c**). Comparison of laminar dynamics of coherence at IG (black) and SG (gray) at 10–25 (left) and 25–40 (right) Hz are also shown (**b** and **d**). Monkey-1; n = 11, 17, and 8 for clusters 1, 2, and 3, respectively. Monkey-2; n = 8, 7, and 5 for clusters 1, 2, and 3, respectively. Note that coherence patterns were different between monkeys. This may be because fewer data points were involved per cluster for each monkey than the number of summed points. Therefore, coherence patterns in each monkey are more variable than those of the summed data points. However, a five-way ANOVA for clusters (1–3), monkeys, layers (IG and SG), periods (cue and delay), and frequencies (10–25 and 25–40 Hz) showed that there was no significant main effect of monkeys (F = 0.00470, P = 0.946). Note that the lack of significance for the monkey effect can result from the lack of statistical power. When the interaction in cluster 1 was examined separately for each monkey, there was a significant interaction in monkey-1 (F = 28.82, P = 0.0003 at 10–25 Hz) and a marginally significant interaction in monkey-2 (F = 5.42, P = 0.053 at 10–25 Hz). These results indicate that laminar rerouting of coherence between the spiking activity in A36 and LFP in TE was observed in both monkeys. (**e**, **f**) Signal content of A36 neurons in each cluster. *, $P < 0.05$; Tukey–Kramer test after one-way ANOVA. Neither a main effect of monkeys nor interaction were significant in all three indices: pair-recall index (PRI), pair-coding index (PCI), and cue-holding index (CHI); two-way ANOVA for monkey and

cluster effects (PRI: F = 2.93, P = 0.09 for monkey effect; F = 0.62, P = 0.54 for interaction; PCI: F = 1.15, P = 0.29 for monkey effect; F = 1.99, P = 0.15 for interaction; CHI: F = 0.083, P = 0.77 for monkey effect; F = 0.80, P = 0.46 for interaction). Therefore, index values were not statistically different between monkeys. Error bars, mean ± s.e.m.

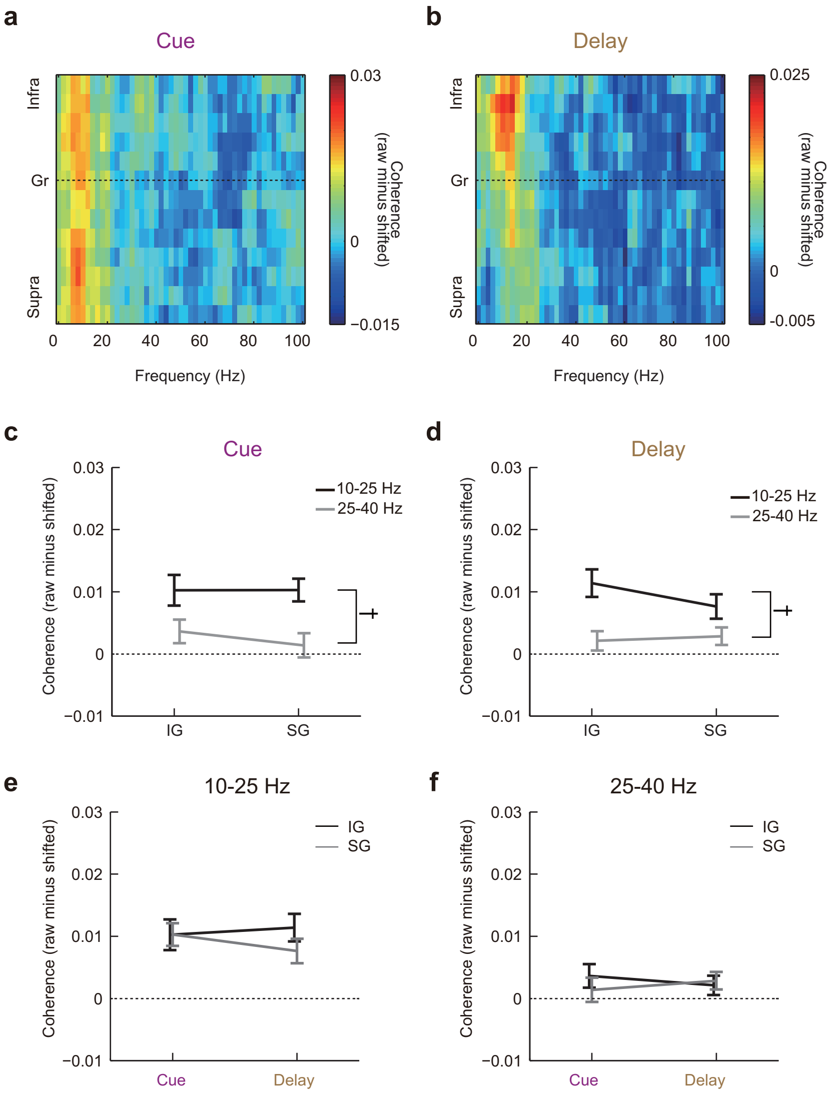

Supplementary Figure 11

Supplementary Figure 11 | Overall, average laminar pattern of coherence. Average laminar patterns of coherence across clusters 1, 2, and 3 are shown for the cue (**a**) and delay period (**b**). (**c**–**f**) The overall coherence in TE did not show a significant difference between layers or periods (two-way ANOVA; 10–25 Hz, $F = 0.12$, $P = 0.73$ for period effect, $F = 0.99$, $P = 0.32$ for layer effect, $F = 1.18$, $P = 0.28$ for interaction; 25–40 Hz, $F = 0.0001$, $P = 0.99$ for period effect, $F = 0.25$, $P = 0.62$ for layer effect, $F = 0.97$, $P = 0.33$ for interaction). These results suggest that, the overall, average coherence did not reflect the average cluster 1 pattern, and rerouting of the laminar flow of information was not a general phenomenon in TE. This indicates that the laminar pattern of coherence in cluster 1 is different from the patterns in clusters 2 and 3. Thus, the laminar rerouting of the inter-areal coherence in cluster 1 could not be extrapolated simply by considering the overall, average coherence in TE. Using the cluster analysis, we were able to reveal that a heterogeneous population of coherence in area TE could be divided into three distinct patterns, each of which involved A36 neurons showing distinct types of mnemonic coding (high PRI in cluster 1 and high PCI in cluster 2), from a heterogeneous coherence population. IG, infragranular layer; SG, supragranular layer. Error bars, mean ± s.e.m.

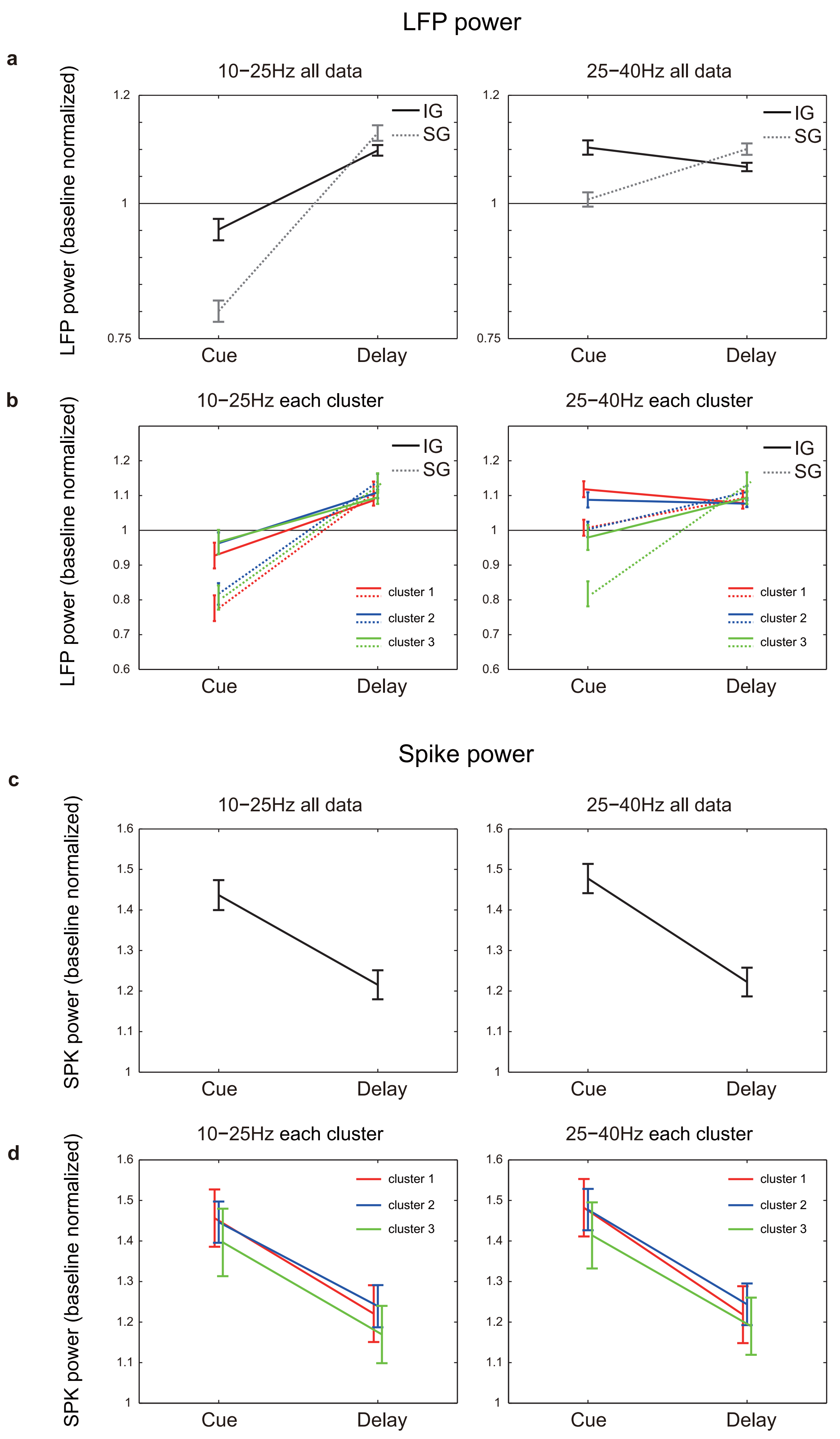

Supplementary Figure 12

Supplementary Figure 12 | Local field potential (LFP) power and spike power. Powers of LFP (**a**, **b**) and spiking activity (**c**, **d**) are shown across task time periods. The LFP power showed a significant period effect (**b**, three-way ANOVA for period effect, layer effect, and cluster effect: $F = 366.98$, $P < 0.0001$ at 10–25 Hz; $F = 4.44$, $P < 0.040$ at 25–40 Hz). The spike power also showed a significant period effect (**d**, two-way ANOVA for period effect and cluster effect: $F = 48.73$, $P < 0.0001$ at 10–25 Hz; $F = 62.08$, $P < 0.0001$ at 25–40 Hz). However, neither LFP nor spike powers differed across clusters (**b**, LFP power at 10–25 Hz: $F = 0.49$, $P = 0.62$ for cluster effect; $F = 0.013$, $P = 0.99$ for cluster × layer effects; $F = 0.23$, $P = 0.79$ for cluster × period effects; $F = 0.11$, $P = 0.89$ for cluster × layer × period effects; LFP power at 25–40 Hz: $F = 0.13$, $P = 0.88$ for cluster effect; $F = 1.45$, $P = 0.0.24$ for cluster × layer effects; $F = 1.56$, $P = 0.22$ for cluster × period effects; $F = 0.37$, $P = 0.69$ for cluster × layer × period effects; **d**, spike power at 10–25 Hz: $F = 0.23$, $P = 0.79$ for cluster effect; $F = 0.080$, $P = 0.92$ for cluster × period effects; spike power at 25–40 Hz: $F = 0.049$, $P = 0.95$ for cluster effect; $F = 0.19$, $P = 0.83$ for cluster × period effects). Therefore, as theoretically expected, it is unlikely that either LFP power or spike power influenced spike-field measures, which differed between clusters. IG, infragranular layer; SG, supragranular layer. Error bars, mean ± s.e.m.

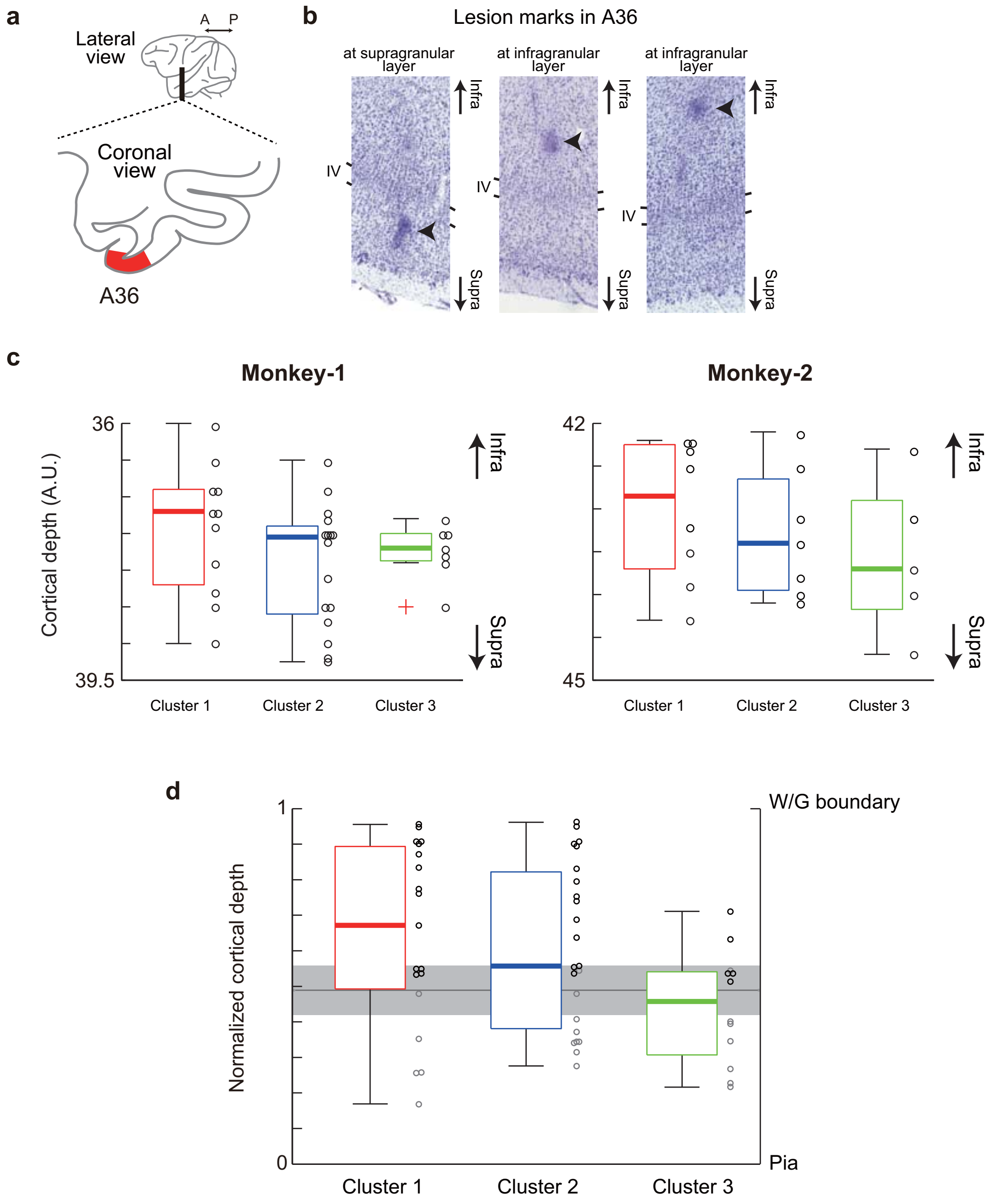

Supplementary Figure 13

Supplementary Figure 13 | Cortical depth of A36 neurons. (**a**) Cortical location of A36. (**b**) Examples of lesion marks in A36. The lesion marks were used for estimating the cortical location of A36 neurons in the histological sections. (**c**) Cortical depth of A36 neurons in each cluster for monkey-1 (left) and monkey-2 (right). See Methods for details. Thick lines depict the median. Circles depict the cortical depth of each neuron. Relative cortical depth of A36 neurons across clusters is similar between monkeys. (**d**) Normalized cortical depth of A36 neurons. The location of the pia mater and white/gray matter (W/G) boundary is equal to zero and one, respectively. Black and gray circles depict location of neurons in the infragranular and supragranular layers, respectively. Gray shading depicts average ± standard deviation of normalized cortical depth of center of the granular layer determined by histological images. See Methods for detailed procedures. Differences in normalized cortical depth of A36 neurons across clusters were marginally significant (χ^2 = 5.81, P = 0.0548, Kruskal–Wallis test). Note that the locations of A36 neurons in cluster 1 were significantly deeper than the average normalized cortical depth of the granular layer (z = 2.37, P = 0.0352, Wilcoxon's signed-rank test corrected for multiple comparisons with Bonferroni's method), while those in cluster 2 were not (P = 0.0616).

LFP γ-power (cue period)

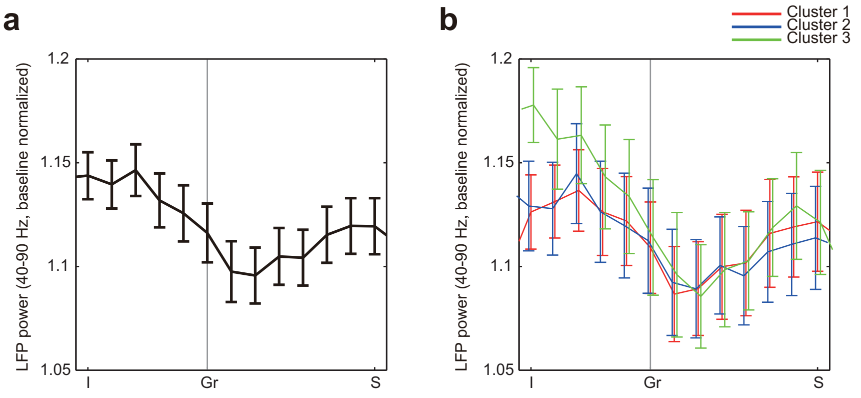

LFP γ-power (delay period)

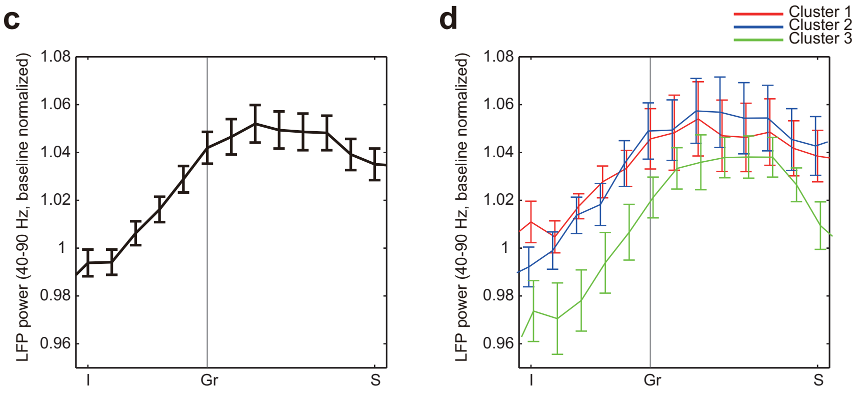

Supplementary Figure 14

Supplementary Figure 14 | Population depth profile of γ-power. (**a**, **b**) Mean γ-power (± s.e.m.) across all datasets (**a**) and for each cluster (**b**) during the cue period. The γ-power was normalized against γ-power during the fixation period. Two-way ANOVA for channels and clusters showed the main effect for channels to be significant ($F = 9.22$, $P < 0.001$), but there was no significant difference between clusters ($F = 0.787$, $P = 0.465$) or interaction between channels and clusters ($F = 0.386$, $P = 0.997$). (**c, d**) Mean γ-power (± s.e.m.) across all datasets (**c**) and for each cluster (**d**) during the delay period. Two-way ANOVA for channels and clusters showed the main effect for channels to be significant ($F = 9.98$, $P < 0.001$), but there was no significant difference between clusters ($F = 1.05$, $P = 0.365$) or interaction between channels and clusters ($F = 1.18$, $P = 0.256$). Error bars, mean ± s.e.m.

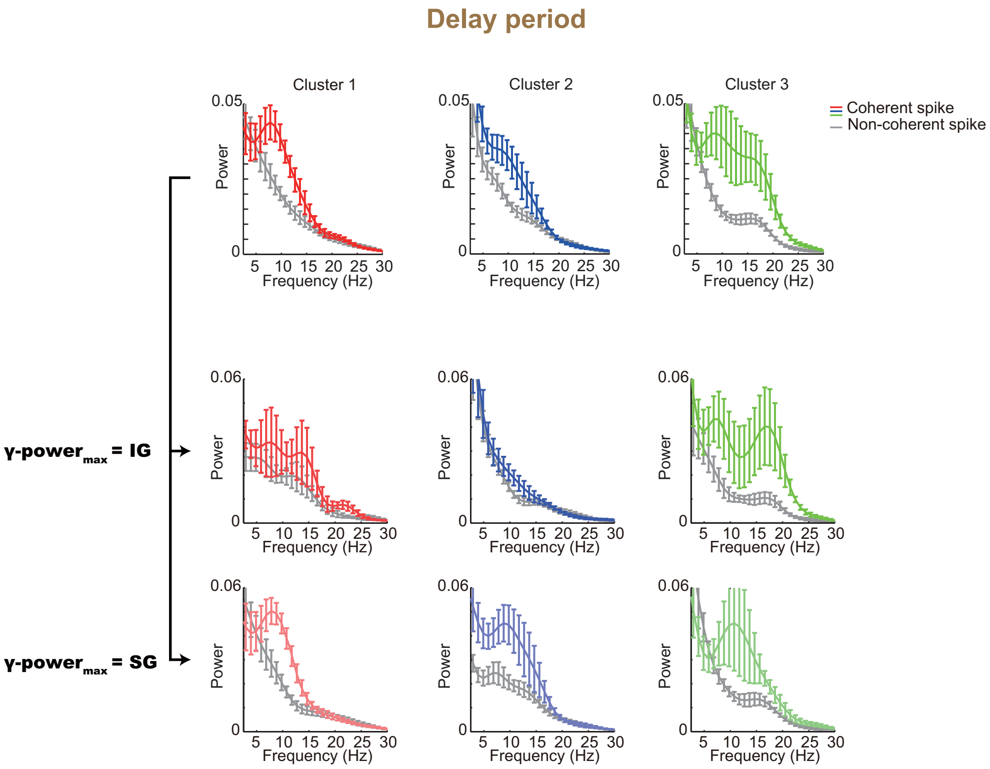

Supplementary Figure 15

Supplementary Figure 15 | Differential increase in STAγ power depending on channels showing maximum γ-power at the infragranular (IG) and supragranular (SG) layer during the delay period. Top, original STAγ power (same as in Fig. 4f). Middle, STAγ power when maximum γ-power was observed at IG. Bottom, STAγ power when maximum γ-power was observed at SG. Error bars, mean ± s.e.m.

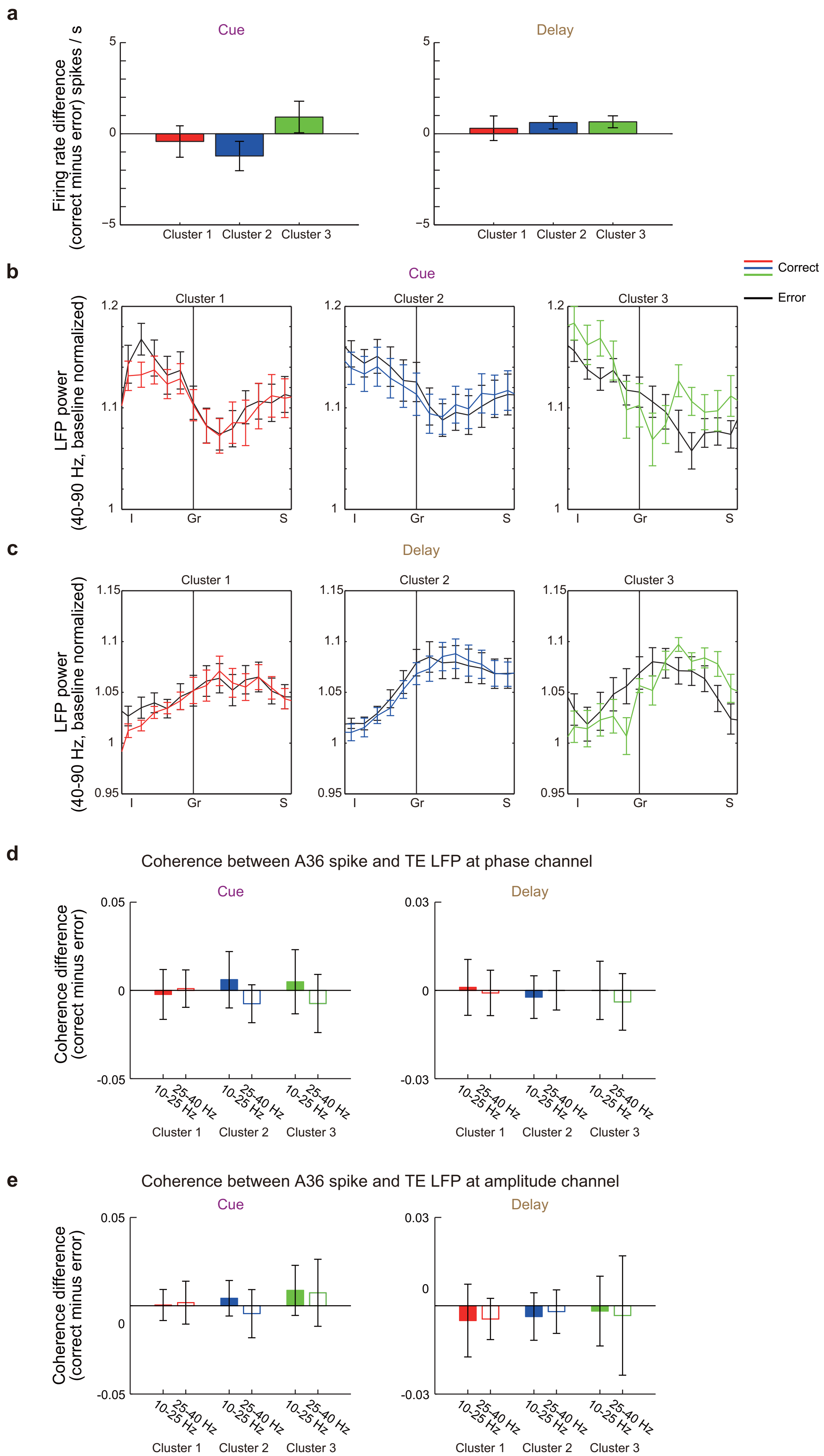

Supplementary Figure 16

Supplementary Figure 16 | Behavioral relevance of neuronal measures. Note that the number of correct trials was reduced to match that of error trials as in the STAγ (see Methods). (**a**) Comparison of the firing rate of A36 neurons between correct and error trials (paired *t*-test, $t = -0.0485$, $P = 0.97$). The firing rate of A36 neurons was not significantly different between correct and error trials during the cue period [two-way ANOVA for trial types (correct or error) and clusters: $F = 0.0399$, $P = 0.842$ for trial type effect] and delay period (two-way ANOVA: $F = 0.153$, $P = 0.697$ for trial type effect). (**b**, **c**) Comparison of the layer specificity of TE γ-power between correct and error trials during the cue period (**b**) and delay period (**c**). The layer specificity of TE γ-power was not significantly different between correct and error trials during the cue period [two-way ANOVA for trial types (correct or error) and clusters: $F = 0.189$, $P = 0.668$ for trial type effect] and delay period (two-way ANOVA: $F = 0.0927$, $P = 0.763$ for trial type effect). (**d**–**e**) Comparison of the coherence between correct and error trials. Shown are coherence between A36 spiking activity and TE LFP at the channel showing maximum coherence (phase channel) (**d**) and coherence between A36 spiking activity and TE LFP at the channel showing maximum γ power (amplitude channel) (**e**). Note that LFP at the phase channel was used to extract coherence spikes in STAγ, and that LFP at the amplitude channel was used to calculate spike-triggered average in STAγ. The three-way ANOVA for cluster (1, 2, 3), frequency (10–25/25–40 Hz), and monkey's behavior (correct/error) did not reveal any significant main effect of behavior or interaction between behavior and other factors involved in the coherence between A36 spiking activity and TE LFP at the phase channel (**d** left; cue period: $F = 0.0073$, $P = 0.93$ for main effect of behavior; $F = 0.0008$, $P = 0.99$ for

interaction between behavior and cluster; $F = 0.43$, $P = 0.51$ for interaction between behavior and frequency; $F = 0.22$, $P = 0.80$ for interaction among three factors) (**d** right; delay period: $F = 0.042$, $P = 0.84$ for main effect of behavior; $F = 0.018$, $P = 0.98$ for interaction between behavior and cluster; $F = 0.042$, $P = 0.84$ for interaction between behavior and frequency; $F = 0.029$, $P = 0.97$ for interaction among three factors). A significant difference in coherence between correct and error trials was also not observed in the coherence between A36 spiking activity and TE LFP at the amplitude channel during the cue period (**e** left; $F = 0.23$, $P = 0.63$ for main effect of behavior; $F = 0.15$, $P = 0.86$ for interaction between behavior and cluster; $F = 0.057$, $P = 0.81$ for interaction between behavior and frequency; $F = 0.077$, $P = 0.93$ for interaction among three factors) as well as during the delay period (**e** right; $F = 0.46$, $P = 0.50$ for main effect of behavior; $F = 0.033$, $P = 0.97$ for interaction between behavior and cluster; $F = 0.0004$, $P = 0.98$ for interaction between behavior and frequency; $F = 0.0041$, $P = 0.99$ for interaction among three factors). These results indicate that interaction between inter-areal and within-areal signals (STAγ) is highly relevant with monkeys' behavior, even in the case where the inter-areal coherence does not necessarily reflect the behavior. Error bars, mean ± s.e.m.

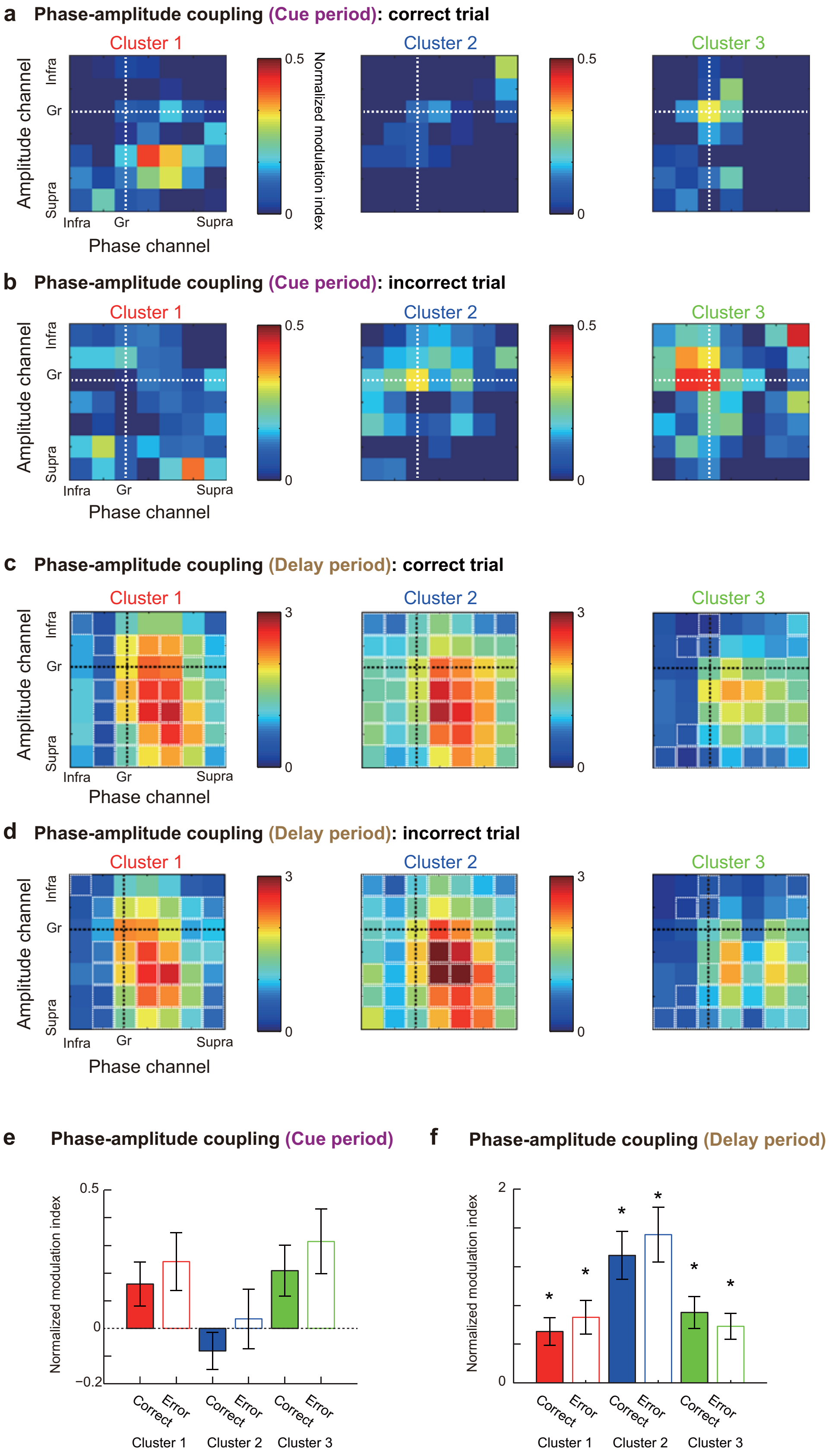

Supplementary Figure 17

Supplementary Figure 17 | Phase-amplitude-coupling (PAC) within and between layers in TE. (**a–d**) Comprehensive PAC analysis within and between channels in TE during the cue and delay period, in correct and error trials separately. Normalized modulation index between low-frequency local field potential (LFP) (13–23 Hz for cue period and 5–18 Hz for delay period) at a phase channel (x-axis) and high-frequency LFP (40–90 Hz) at an amplitude channel (y-axis) is color-coded (See Methods for details). A white dotted square depicts a significant PAC value ($P < 0.05$ false discovery rate). For the cue period, PAC analysis did not show any significant signal patterns in any cluster, irrespective of the monkey's performance (**a–b**). In contrast, during the delay period, PAC analysis showed significant signal patterns in all clusters in both correct and error trials (**c–d**). (**e–f**) PAC analysis between the particular phase and amplitude channels; the phase channel is the channel of the inter-areal coherence in STAγ, and the amplitude channel is the channel showing the maximum γ power. Conversely, in STAγ, A36 spikes coherent with TE LFP at the phase channel were extracted to calculate the spike-triggered average of the TE γ-power at the amplitude channel. Error bars, mean ± s.e.m. The results showed that there was no significant PAC during the cue period. In contrast, during the delay period, PAC values were significant in all clusters in both correct and error trials (*t*-test, $P < 0.05$ corrected by Bonferroni's method), while PAC values differed slightly across clusters (two-way ANOVA for clusters and monkey's performance: $F = 4.47$, $P = 0.015$). It is noteworthy that PAC in the correct trials was not significantly different from that in error trials during both the cue and delay periods ($F = 1.07$, $P = 0.30$ for performance effect during the cue period; $F = 0.022$, $P = 0.88$ for performance effect during the delay period).

These PAC results are different from the results obtained with STAγ. First, during the cue period, STAγ in cluster 1 showed a low-frequency periodic increase in γ-power in correct trials (Fig. 4g). Second, during the delay period, STAγ was significant only in correct trials but not in error trials in all clusters. The difference between the results in PAC and in STAγ indicates that the STAγ used in the present study captures the time relationship between spiking activity in A36 and LFP in TE, i.e., across-areal interactions; these are not captured by PAC within and between layers in TE. Error bars, mean ± s.e.m.
